# Supplementary material for: TMPRSS2-mediated coronavirus spike activation and inhibition
Source: Nat Struct Mol Biol. 2026 Apr 28;33(5):810–23. doi: 10.1038/s41594-026-01801-y (PMC13186702; doi:10.1038/s41594-026-01801-y)

nature structural & molecular biology

Supplementary information https://doi.org/10.1038/s41594-026-01801-y

TMPRSS2-mediated coronavirus spike activation and inhibition

In the format provided by the authors and unedited

Supplementary information

Supplementary information file:

Supplementary Figures 1-20

Supplementary Tables 1-3

Source Data for Supplementary Figures

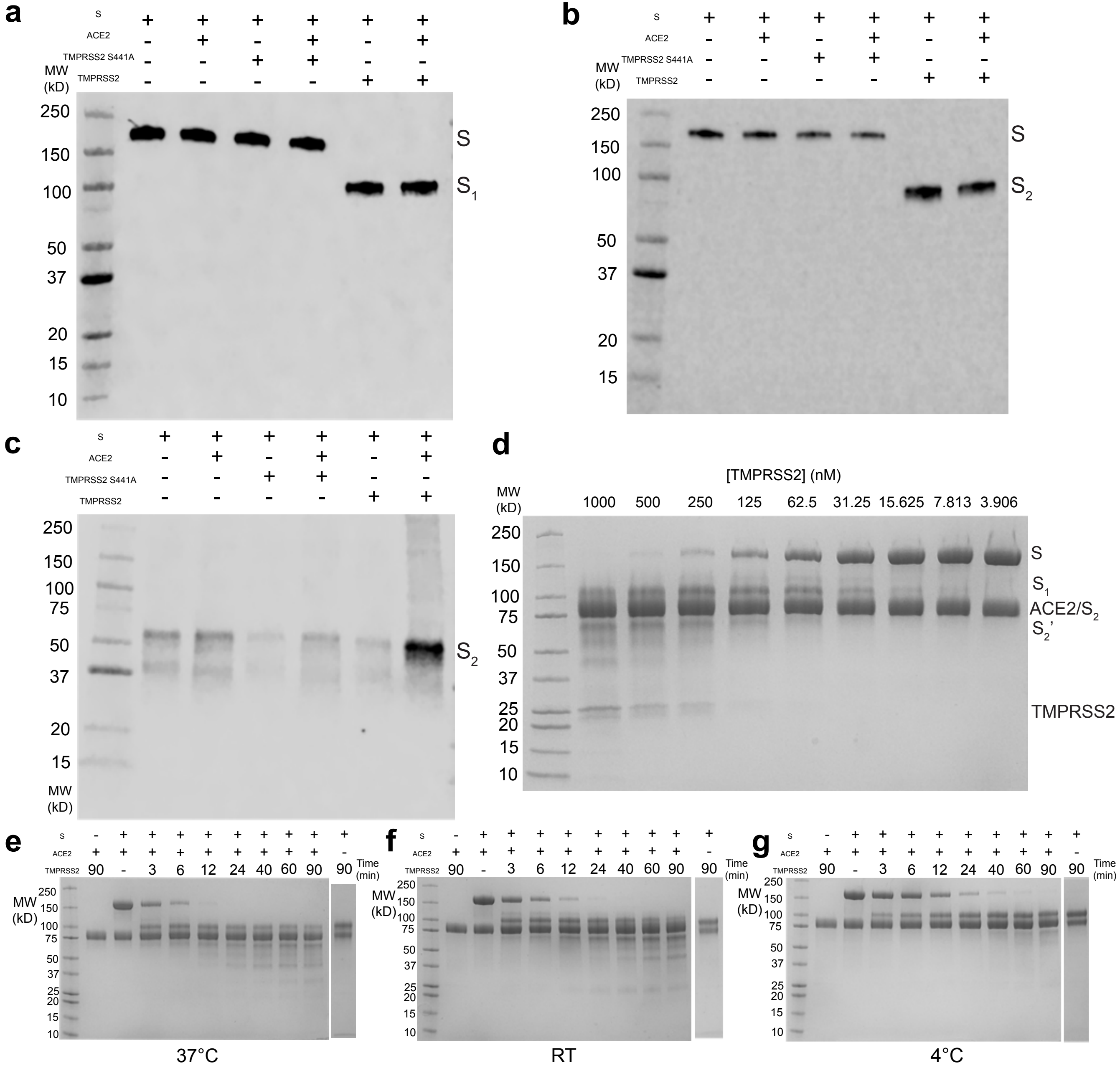

a
S
ACE2
TMPRSS2 S441A
TMPRSS2
MW (kD)
250
150
100
50
37
20
15
10
S
S1
b
S
ACE2
TMPRSS2 S441A
TMPRSS2
MW (kD)
250
150
100
50
37
20
15
S
S2
c
S
ACE2
TMPRSS2 S441A
TMPRSS2
250
150
100
75
50
37
20
15
MW (kD)
S2
d
[TMPRSS2] (nM)
MW (kD)
1000
500
250
125
62.5
31.25
15.625
7.813
3.906
250
150
100
75
50
37
25
20
15
10
S
S1
ACE2/S2
S2'
TMPRSS2
e
S
ACE2
TMPRSS2
90
3
6
12
24
40
60
90
90
Time (min)
MW (kD)
250
150
100
75
50
37
25
20
15
10
37°C
f
S
ACE2
TMPRSS2
90
3
6
12
24
40
60
90
90
Time (min)
MW (kD)
250
150
100
75
50
37
25
20
15
10
RT
g
S
ACE2
TMPRSS2
90
3
6
12
24
40
60
90
90
Time (min)
MW (kD)
250
150
100
75
50
37
25
20
15
10
4°C

Supplementary Figure 1. SDS-PAGE and western blot analysis of TMPRSS2-mediated cleavage of SARS-CoV-2 S_{ecto} for the first biological replicate. a-b, Western blot analysis of SARS-CoV-2 S_{ecto} at a concentration of 0.65 µM incubated (or not) with 2 µM of the monomeric human ACE2 (peptidase) ectodomain for 5 minutes prior to incubation (or not) with the TMPRSS2 ectodomain at a concentration of 0.2 µM for 45 min (as described above each lane). All steps were carried out at 4˚C. Detection used an anti-SARS-CoV-2 RBD (a) or the S_2' site-directed 76E1 (b) primary antibodies. **c,** Western blot analysis of SARS-CoV-2 S_{ecto} incubated or not with ACE2 and/or TMPRSS2 in conditions identical to panels a-b (as described above each lane) prior to digestion with Proteinase K at a final concentration of 10µg/mL and a final SARS-CoV-2 S_{ecto} concentration of 330nM for 30 min at 4°C detected with a polyclonal S_2 primary antibody. **d,** SDS-PAGE analysis of SARS-CoV-2 S_{ecto} incubated with the monomeric human ACE2 (peptidase) ectodomain at a 1:3 molar ratio for 5 minutes prior to addition of the TMPRSS2 ectodomain at various concentrations (yielding a final SARS-CoV-2 S_{ecto} concentration of 0.625 µM) for 15 min. All steps were carried out at room temperature. These data were used to determine that S_2' cleavage can be observed at a TMPRSS2 concentration range of 125-250nM with minimal nonspecific cleavage. **e-g,** SDS-PAGE analysis of the SARS-CoV-2 S_{ecto} at a final concentration of 1.17 µM incubated with the monomeric human ACE2 (peptidase) ectodomain at a 1:3 molar ratio for 5 minutes prior to addition of the TMPRSS2 ectodomain at a concentration of 0.1 µM. All steps were carried out at 37°C (e), room temperature (f), or 4°C (g) before stopping the reaction at various time points (as described above each lane) by denaturation.

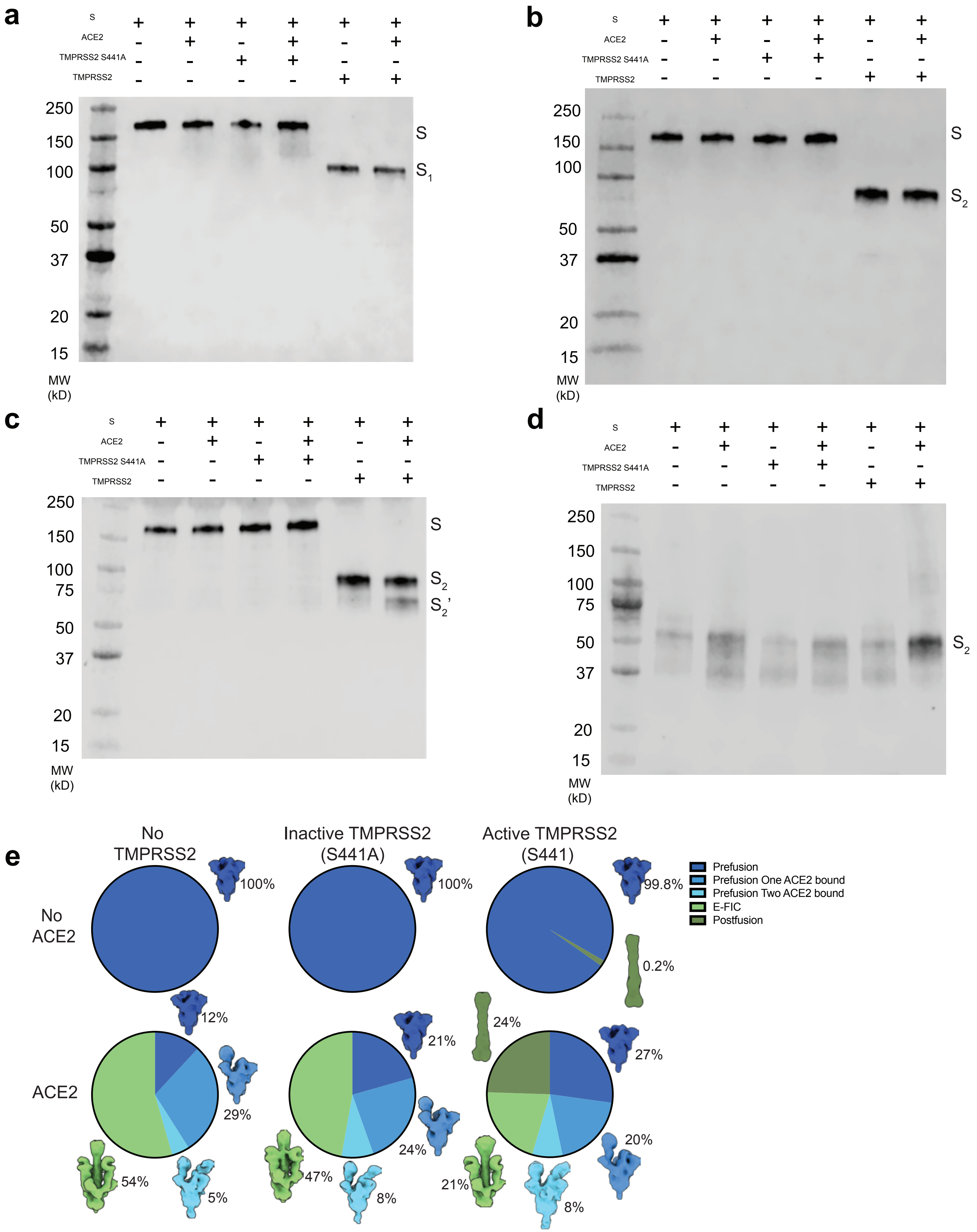
a
S
ACE2
TMPRSS2 S441A
TMPRSS2
250
150
100
50
37
20
15
MW (kD)
S
S1
b
S
ACE2
TMPRSS2 S441A
TMPRSS2
250
150
100
50
37
20
15
MW (kD)
S
S2
c
S
ACE2
TMPRSS2 S441A
TMPRSS2
250
150
100
75
50
37
20
15
MW (kD)
S
S2
S2'
d
S
ACE2
TMPRSS2 S441A
TMPRSS2
250
150
100
75
50
37
20
15
MW (kD)
S2
e
No TMPRSS2
Inactive TMPRSS2 (S441A)
Active TMPRSS2 (S441)
No ACE2
ACE2
100%
100%
99.8%
0.2%
12%
29%
5%
54%
21%
24%
8%
47%
24%
27%
20%
8%
21%
Prefusion
Prefusion One ACE2 bound
Prefusion Two ACE2 bound
E-FIC
Postfusion

Supplementary Figure 2. Western blot analysis of TMPRSS2-mediated cleavage and conformational distribution of SARS-CoV-2 S_{ecto} for the second biological replicate. a-c, Western blot analysis of SARS-CoV-2 S_{ecto} at a concentration of 0.65 μM incubated (or not) with 2 μM of the monomeric human ACE2 (peptidase) ectodomain for 5 minutes prior to incubation (or not) with the TMPRSS2 ectodomain at a concentration of 0.2 μM for 45 min (as described above each lane). All steps were carried out at 4˚C. Detection used an anti-SARS-CoV-2 RBD (a), the S_2' site-directed 76E1 (b) or the stem helix-directed B6 (c) primary antibodies. **d,** Western blot analysis of SARS-CoV-2 S_{ecto} incubated or not with ACE2 and/or TMPRSS2 in conditions identical to panels a-c (as described above each lane) prior to digestion with Proteinase K at a final concentration of 10μg/mL and a final SARS-CoV-2 S_{ecto} concentration of 330nM for 30 min at 4°C detected with a polyclonal S_2 primary antibody. **e,** Second biological replicate of single particle EM analysis of conformations detected for negatively stained SARS-CoV-2 S_{ecto} at a concentration of 0.65 μM incubated (or not) with 2 μM of the monomeric human ACE2 (peptidase) ectodomain for 5 minutes prior to incubation (or not) with the TMPRSS2 ectodomain at a concentration of 0.2 μM for 45 min. All steps were carried out at 4˚C. Pie charts show the distribution of selected particles in each conformation and corresponding 3D reconstructions for the indicated datasets.

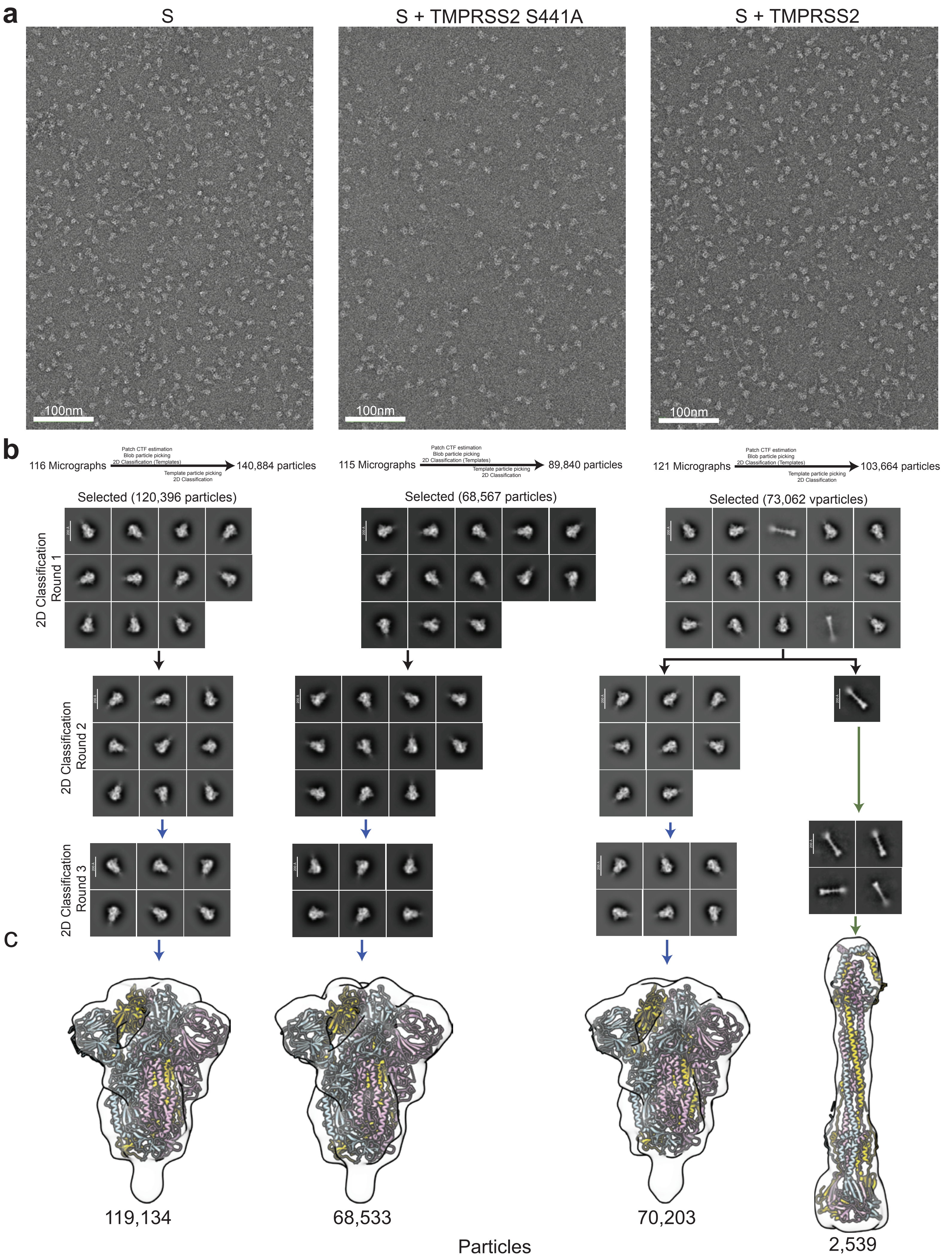
a
S
S + TMPRSS2 S441A
S + TMPRSS2
100nm
100nm
100nm
b
116 Micrographs
Patch CTF estimation
Blob particle picking
2D Classification (Templates)
Template particle picking
2D Classification
140,884 particles
115 Micrographs
89,840 particles
121 Micrographs
103,664 particles
Selected (120,396 particles)
Selected (68,567 particles)
Selected (73,062 vparticles)
2D Classification Round 1
2D Classification Round 2
2D Classification Round 3
c
119,134
68,533
70,203
2,539
Particles

Supplementary Figure 3. EM data processing workflow for SARS-CoV-2 S_{ecto} in the presence or absence of TMPRSS2 for the first biological replicate. a, Representative electron micrographs for negatively stained apo SARS-CoV-2 S_{ecto}, SARS-CoV-2 S_{ecto} incubated with 0.2 µM of S441A (inactive) TMPRSS2 ectodomain, or with 0.2 µM of S441 (active) TMPRSS2 ectodomain for 45 minutes at 4°C in the absence of ACE2. The final SARS-CoV-2 S_{ecto} concentration was 0.65 µM. **b,** Iterative 2D classification of the particle images for the corresponding datasets. **c,** 3D reconstructions obtained with the subset of particles selected for each conformation shown as semi-transparent grey surfaces docked with previously determined structures for prefusion S (PDB 7K43) and postfusion S (PDB 8FDW) rendered as ribbons.

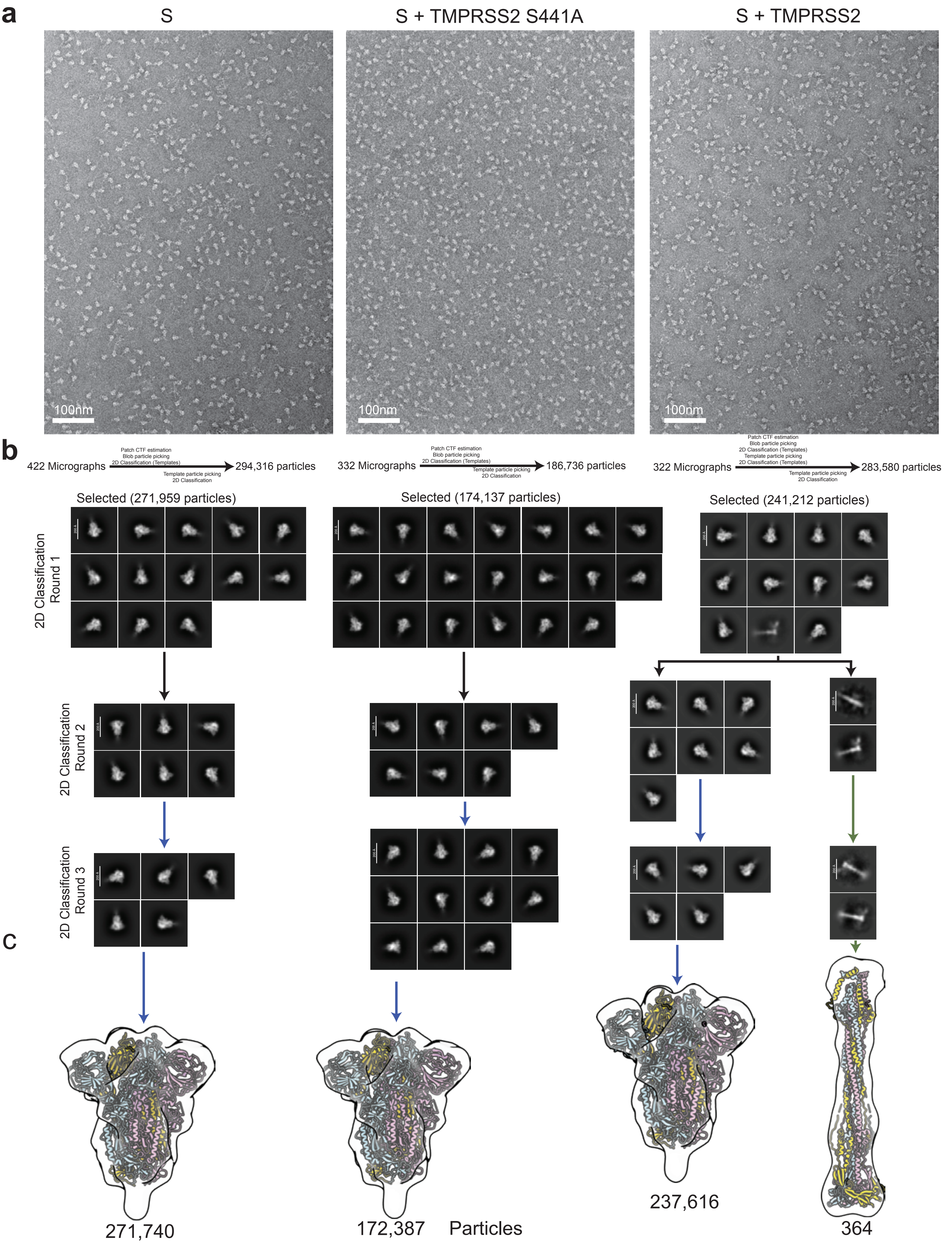

a
S
S + TMPRSS2 S441A
S + TMPRSS2
100nm
100nm
100nm
b
422 Micrographs
Patch CTF estimation
Blob particle picking
2D Classification (Templates)
Template particle picking
2D Classification
294,316 particles
332 Micrographs
Patch CTF estimation
Blob particle picking
2D Classification (Templates)
Template particle picking
2D Classification
186,736 particles
322 Micrographs
Patch CTF estimation
Blob particle picking
2D Classification (Templates)
Template particle picking
2D Classification (Templates)
Template particle picking
2D Classification
283,580 particles
Selected (271,959 particles)
Selected (174,137 particles)
Selected (241,212 particles)
2D Classification Round 1
2D Classification Round 2
2D Classification Round 3
c
271,740
172,387
Particles
237,616
364

Supplementary Figure 4. EM data processing workflow of SARS-CoV-2 S_{ecto} in the presence or absence of TMPRSS2 for the second biological replicate. a, Representative electron micrographs for negatively stained apo SARS-CoV-2 S_{ecto}, SARS-CoV-2 S_{ecto} incubated with 0.2 µM of S441A (inactive) TMPRSS2 ectodomain, or with 0.2 µM of S441 (active) TMPRSS2 ectodomain for 45 minutes at 4°C in the absence of ACE2. The final SARS-CoV-2 S_{ecto} concentration was 0.65 µM. **b,** Iterative 2D classification of the particle images for the corresponding datasets. **c,** 3D reconstructions obtained with the subset of particles selected for each conformation shown as semi-transparent grey surfaces docked with previously determined structures for prefusion S (PDB 7K43) and postfusion S (PDB 8FDW) rendered as ribbons.

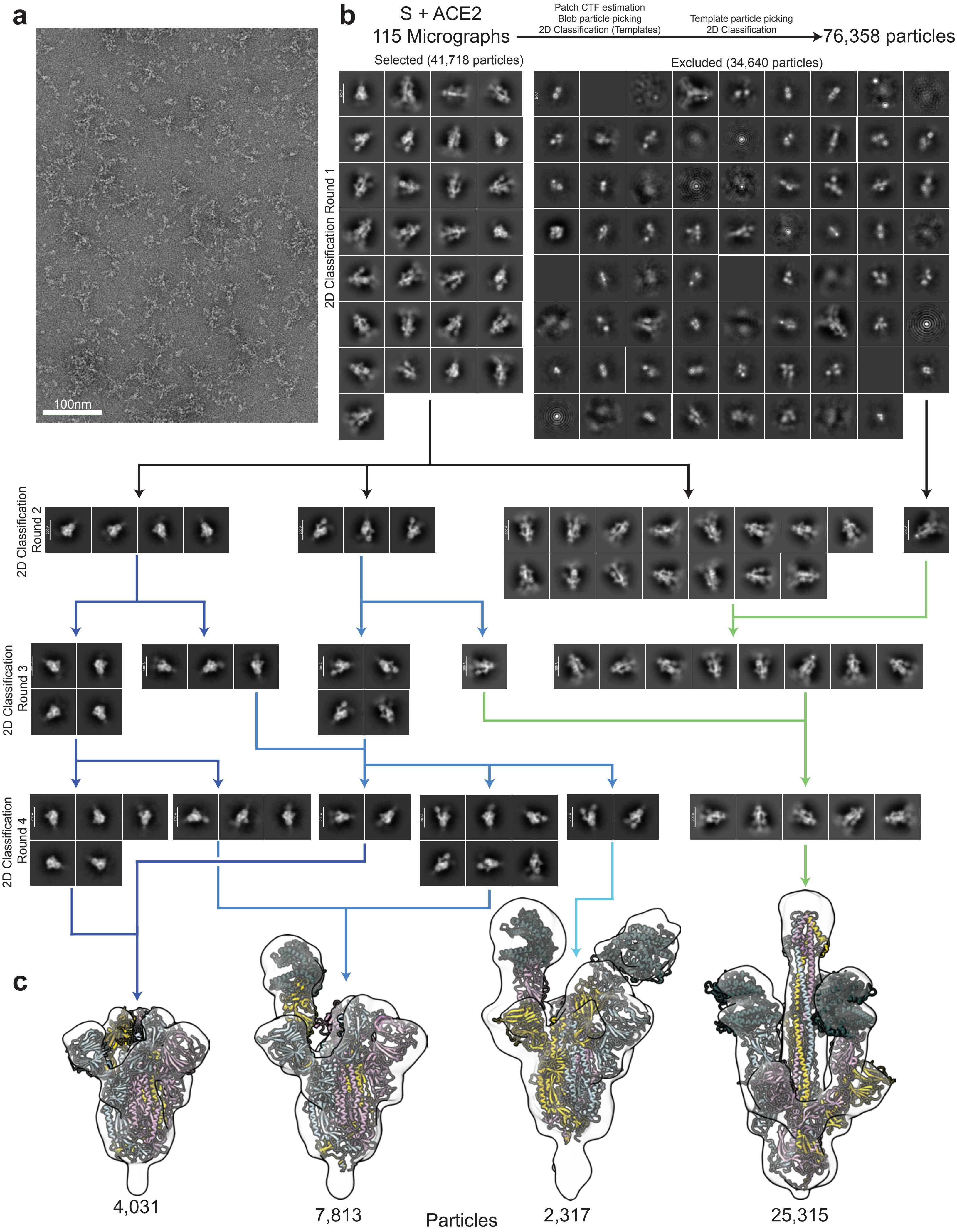

a
100nm
b
S + ACE2
115 Micrographs
Patch CTF estimation
Blob particle picking
2D Classification (Templates)
Template particle picking
2D Classification
76,358 particles
Selected (41,718 particles)
Excluded (34,640 particles)
2D Classification Round 1
2D Classification Round 2
2D Classification Round 3
2D Classification Round 4
c
4,031
7,813
Particles
2,317
25,315

Supplementary Figure 5. EM data processing workflow for SARS-CoV-2 S_{ecto} in the presence of ACE2 for the first biological replicate. a, Representative electron micrograph for negatively stained SARS-CoV-2 S_{ecto} at 0.65 μM incubated with the monomeric ACE2 (peptidase) ectodomain at a 1:3 molar ratio for 45 minutes at 4˚C. **b,** Iterative 2D classification of the particle images leading to identification of distinct S conformations. **c,** 3D reconstructions obtained with the subset of particles selected for each conformation shown as semi-transparent grey surfaces docked with previously determined structures for prefusion S (PDB 7K43), one ACE2-bound prefusion S (PDB 7A94), two ACE2-bound prefusion S with one RBD-ACE2 region fit independently (PDB 7A97), and E-FIC (PDB 8Z7P) rendered as ribbons.

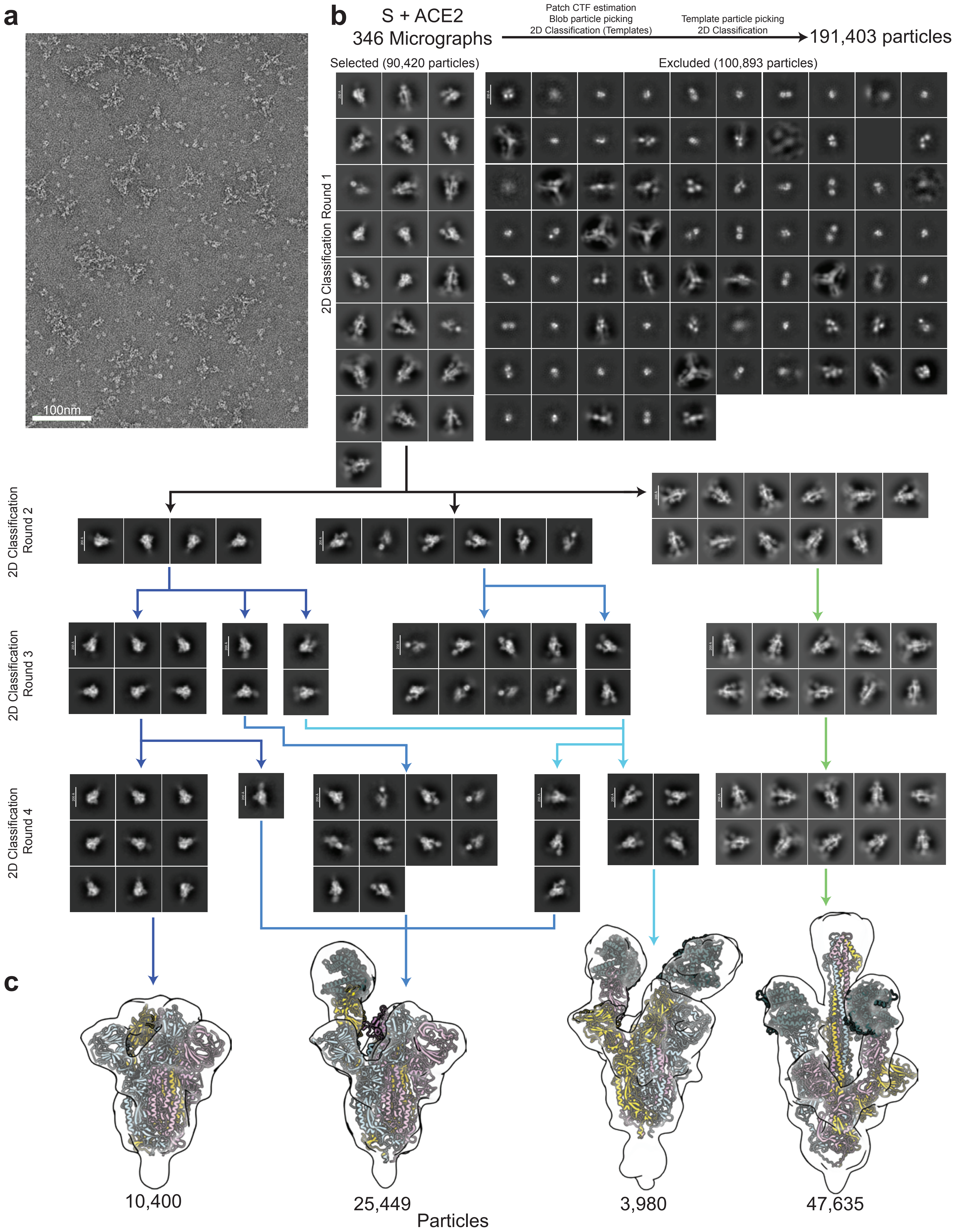

a
100nm
b
S + ACE2
346 Micrographs
Patch CTF estimation
Blob particle picking
2D Classification (Templates)
Template particle picking
2D Classification
191,403 particles
Selected (90,420 particles)
Excluded (100,893 particles)
2D Classification Round 1
2D Classification Round 2
2D Classification Round 3
2D Classification Round 4
c
10,400
25,449
3,980
47,635
Particles

Supplementary Figure 6. EM data processing workflow of SARS-CoV-2 S_{ecto} in the presence of ACE2 for the second biological replicate. a, Representative electron micrograph for negatively stained SARS-CoV-2 S_{ecto} at 0.65 μM incubated with the monomeric ACE2 (peptidase) ectodomain at a 1:3 molar ratio for 45 minutes at 4˚C. **b,** Iterative 2D classification of the particle images leading to identification of distinct S conformations. **c,** 3D reconstructions obtained with the subset of particles selected for each conformation shown as semi-transparent grey surfaces docked with previously determined structures for prefusion S (PDB 7K43), one ACE2-bound prefusion S (PDB 7A94), two ACE2-bound prefusion S with one RBD-ACE2 region fit independently (PDB 7A97), and E-FIC (PDB 8Z7P) rendered as ribbons.

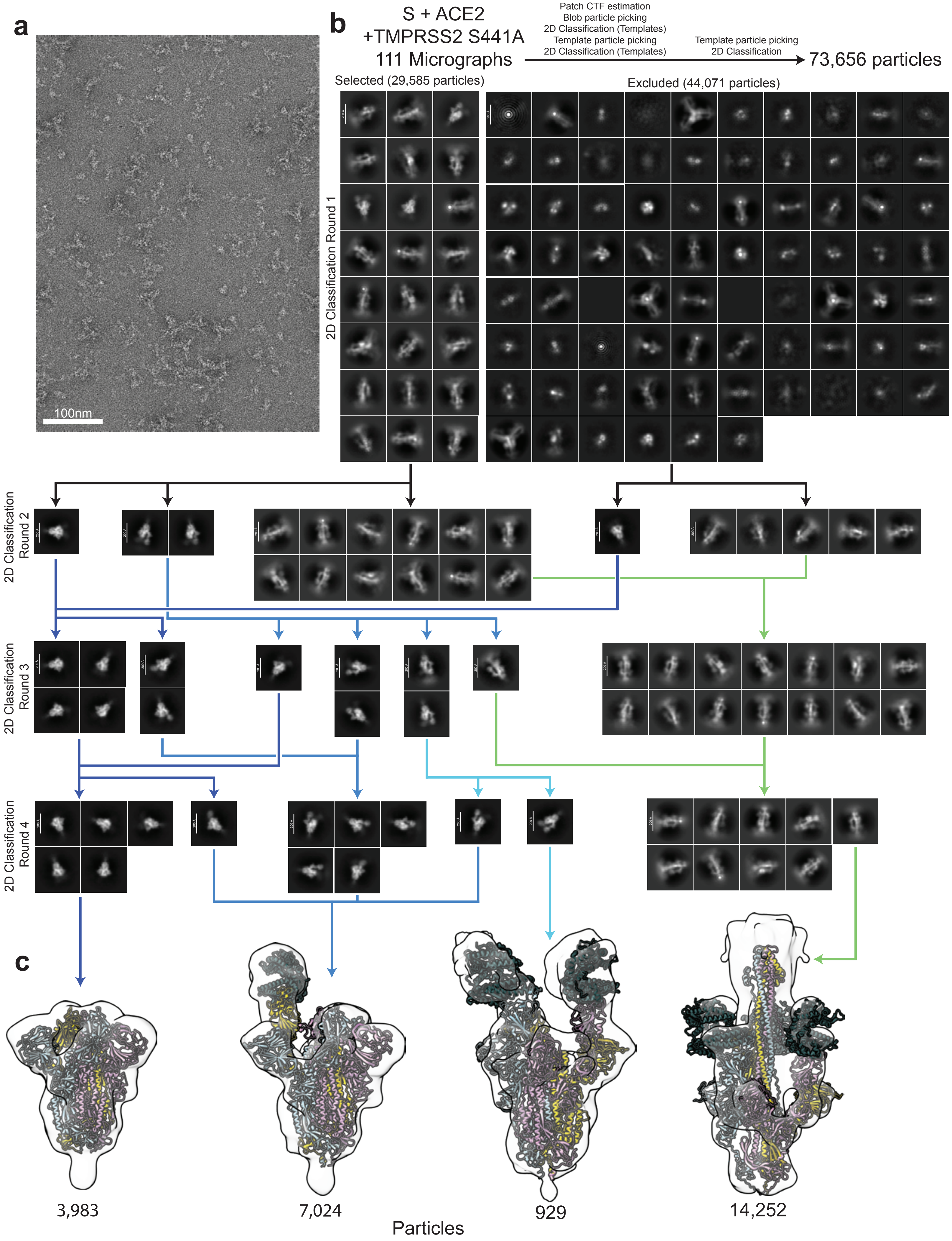

a
100nm
b
S + ACE2
+TMPRSS2 S441A
111 Micrographs
Patch CTF estimation
Blob particle picking
2D Classification (Templates)
Template particle picking
2D Classification (Templates)
Template particle picking
2D Classification
73,656 particles
Selected (29,585 particles)
Excluded (44,071 particles)
2D Classification Round 1
2D Classification Round 2
2D Classification Round 3
2D Classification Round 4
c
3,983
7,024
929
14,252
Particles

Supplementary Figure 7. EM data processing workflow for SARS-CoV-2 S_{ecto} in the presence of ACE2 and inactive TMPRSS2 for the first biological replicate. a, Representative electron micrograph for negatively stained SARS-CoV-2 S_{ecto} at 0.65 μM incubated with the monomeric human ACE2 (peptidase) ectodomain at a 1:3 molar ratio for 5 min followed by 0.2 μM of the S441A TMPRSS2 ectodomain for 45 minutes. All steps were carried out at 4˚C. **b,** Iterative 2D classification of the particle images leading to identification of distinct S conformations. **c,** 3D reconstructions obtained with the subset of particles selected for each conformation shown as semi-transparent grey surfaces docked with previously determined structures for prefusion S (PDB 7K43), one ACE2-bound prefusion S (PDB 7A94), two ACE2-bound prefusion S (PDB 7A97), and E-FIC (PDB 8Z7P) rendered as ribbons.

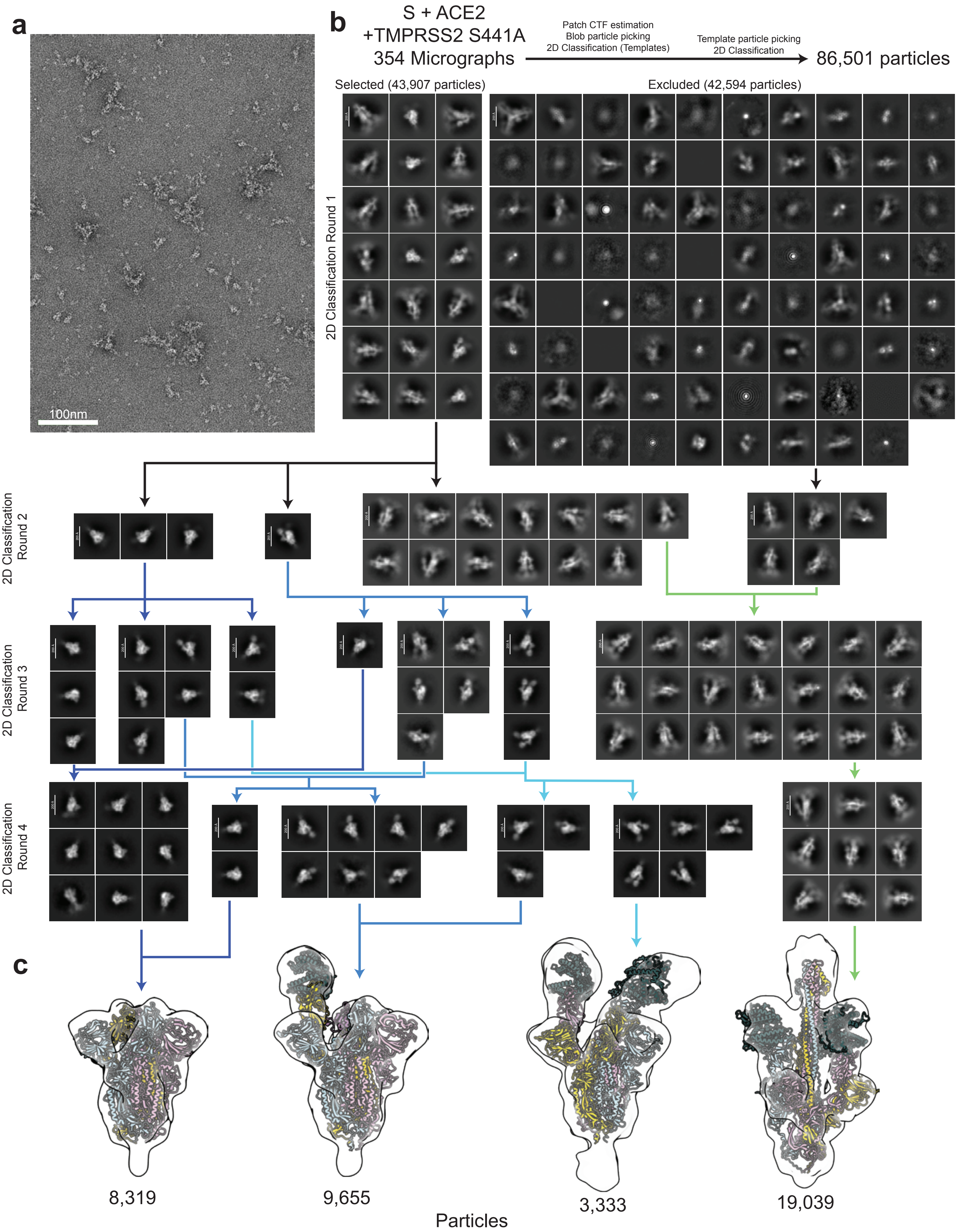
a
100nm
b
S + ACE2
+TMPRSS2 S441A
354 Micrographs
Patch CTF estimation
Blob particle picking
2D Classification (Templates)
Template particle picking
2D Classification
86,501 particles
Selected (43,907 particles)
Excluded (42,594 particles)
2D Classification Round 1
2D Classification Round 2
2D Classification Round 3
2D Classification Round 4
c
8,319
9,655
3,333
19,039
Particles

Supplementary Figure 8. EM data processing workflow of SARS-CoV-2 S_{ecto} in the presence of ACE2 and inactive TMPRSS2 for the second biological replicate. a, Representative electron micrograph for negatively stained SARS-CoV-2 S_{ecto} at 0.65 μM incubated with the monomeric human ACE2 (peptidase) ectodomain at a 1:3 molar ratio for 5 min followed by 0.2 μM of the S441A TMPRSS2 ectodomain for 45 minutes. All steps were carried out at 4˚C. **b,** Iterative 2D classification of the particle images leading to identification of distinct S conformations. **c,** 3D reconstructions obtained with the subset of particles selected for each conformation shown as semi-transparent grey surfaces docked with previously determined structures for prefusion S (PDB 7K43), one ACE2-bound prefusion S (PDB 7A94), two ACE2-bound prefusion S (PDB 7A97), and E-FIC (PDB 8Z7P) rendered as ribbons.

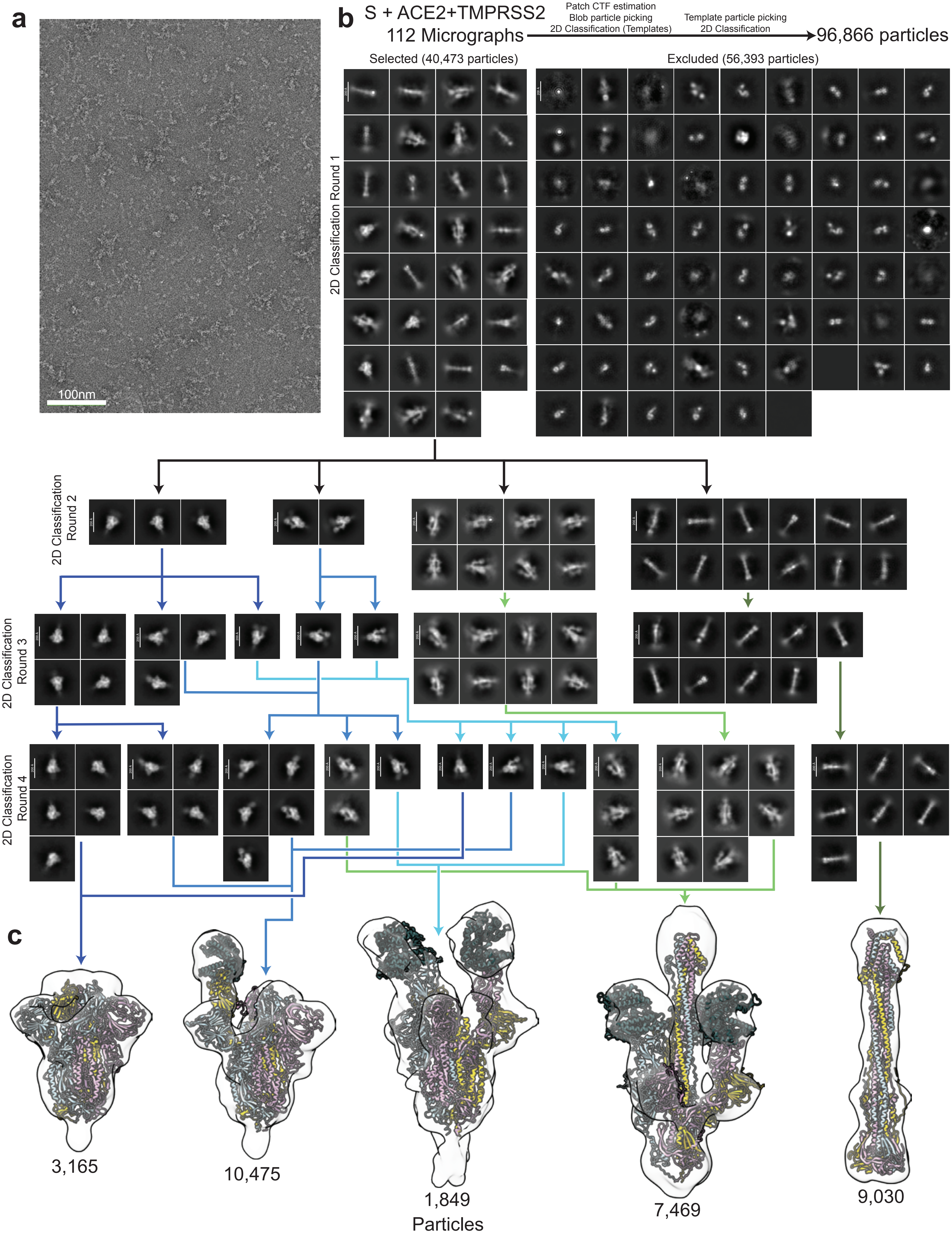

a
100nm
b
S + ACE2+TMPRSS2
112 Micrographs
Patch CTF estimation
Blob particle picking
2D Classification (Templates)
Template particle picking
2D Classification
96,866 particles
Selected (40,473 particles)
Excluded (56,393 particles)
2D Classification Round 1
2D Classification Round 2
2D Classification Round 3
2D Classification Round 4
c
3,165
10,475
1,849
Particles
7,469
9,030

Supplementary Figure 9. EM data processing workflow for SARS-CoV-2 S_{ecto} in the presence of ACE2 and active TMPRSS2 for the first biological replicate. a, Representative electron micrograph for negatively stained SARS-CoV-2 S_{ecto} at 0.65 μM incubated with the monomeric human ACE2 (peptidase) ectodomain at a 1:3 molar ratio for 5 min followed by 0.2 μM of the S441 TMPRSS2 ectodomain for 45 minutes. All steps were carried out at 4˚C. **b,** Iterative 2D classification of the particle images leading to identification of distinct S conformations. **c,** 3D reconstructions obtained with the subset of particles selected for each conformation shown as semi-transparent grey surfaces docked with previously determined structures of prefusion S (PDB 7K43), one ACE2-bound prefusion S (PDB 7A94), two ACE2-bound prefusion S (PDB 7A97), E-FIC (PDB 8Z7P), and postfusion S (PDB 8FDW) rendered as ribbons.

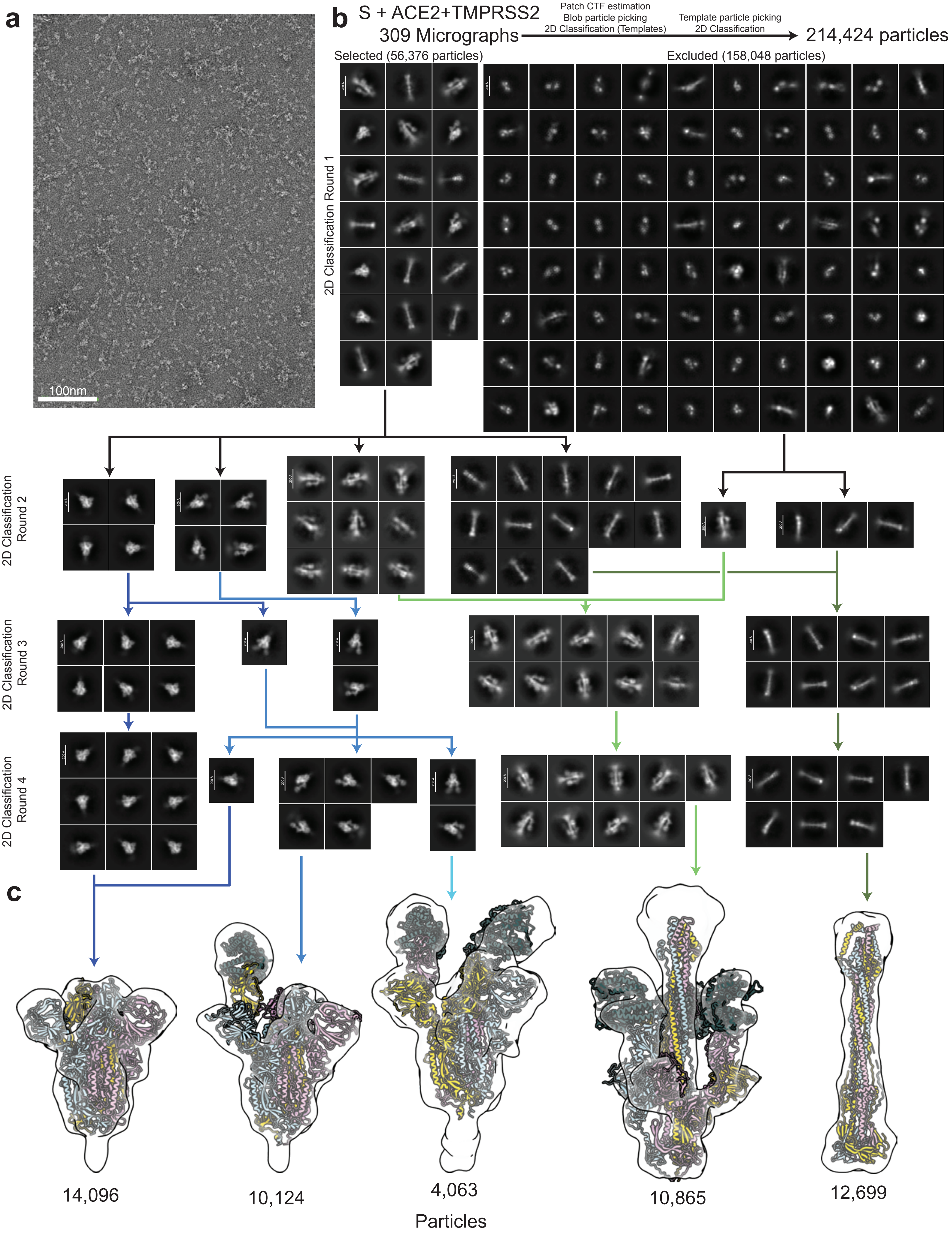
a
100nm
b
S + ACE2+TMPRSS2
309 Micrographs
Patch CTF estimation
Blob particle picking
2D Classification (Templates)
Template particle picking
2D Classification
214,424 particles
Selected (56,376 particles)
Excluded (158,048 particles)
2D Classification Round 1
2D Classification Round 2
2D Classification Round 3
2D Classification Round 4
c
14,096
10,124
4,063
10,865
12,699
Particles

Supplementary Figure 10. EM data processing workflow of SARS-CoV-2 S_{ecto} in the presence of ACE2 and active TMPRSS2 for the second biological replicate. a, Representative electron micrograph for negatively stained SARS-CoV-2 S_{ecto} at 0.65 µM incubated with the monomeric human ACE2 (peptidase) ectodomain at a 1:3 molar ratio for 5 min followed by 0.2 µM of the S441 TMPRSS2 ectodomain for 45 minutes. All steps were carried out at 4˚C. **b,** Iterative 2D classification of the particle images leading to identification of distinct S conformations. **c,** 3D reconstructions obtained with the subset of particles selected for each conformation shown as semi-transparent grey surfaces docked with previously determined structures of prefusion S (PDB 7K43), one ACE2-bound prefusion S (PDB 7A94), two ACE2-bound prefusion S (PDB 7A97), E-FIC (PDB 8Z7P), and postfusion S (PDB 8FDW) rendered as ribbons.

S

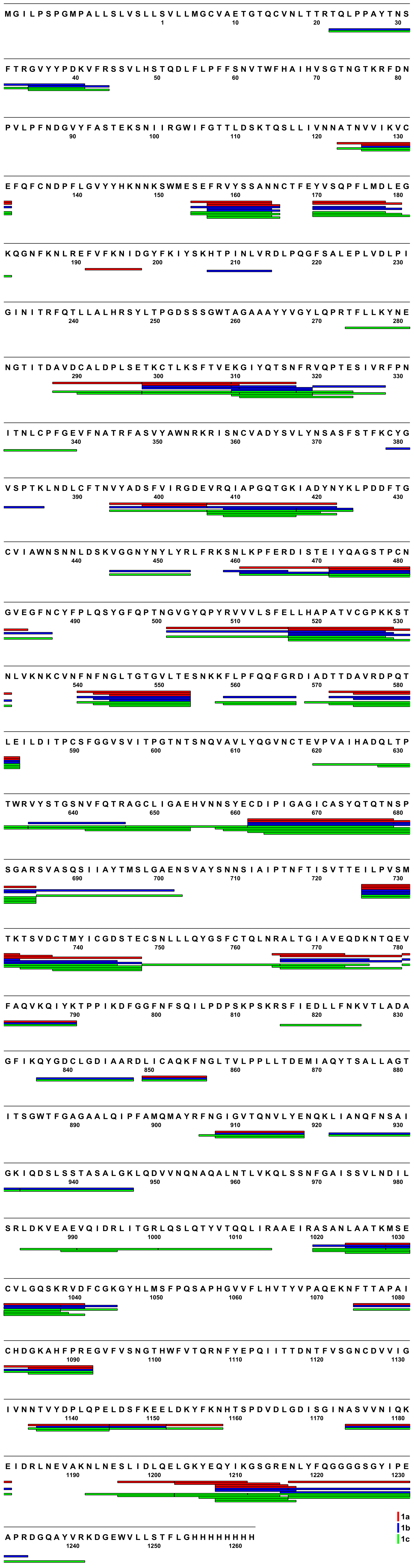
MGILPSPGMPALLSLVSLLSVLLMGCVAETGTQCVNLTTRTQLPPAYTNS
1 10 20 30
FTRGVYYPDKVFRSSVLHSTQDLFLPFFSNVTWFHAIHVSGTNGTKRFDN
40 50 60 70 80
PVLPFNDGVYFASTEKSNIIRGWIFGTTLDSKTQSLLIVNNATNVVIKVC
90 100 110 120 130
EFQFCNDPFLGVYYHKNNKSWMESEFRVYSSANNCTFEYVSQPFLMDLEG
140 150 160 170 180
KQGNFKNLREFVFKNIDGYFKIYSKHTPINLVRDLPQGFSALEPLVDLPI
190 200 210 220 230
GINITRFQTLLALHRSYLTPGDSSSGWTAGAAAYYVGYLQPRTFLLKYNE
240 250 260 270 280
NGTITDAVDCALDPLSETKCTLKSFTVEKGIYQTSNFRVQPTESIVRFPN
290 300 310 320 330
ITNLCPFGEVFNATRFASVYAWNRKRISNCVADYSVLYNSASFSTFKCYG
340 350 360 370 380
VSPTKLNDLCFTNVYADSFVIRGDEVRQIAPGQTGKIADYNYKLPDDFTG
390 400 410 420 430
CVIAWNSNNLDSKVGGNYNYLYRLFRKSNLKPFERDISTEIYQAGSTPCN
440 450 460 470 480
GVEGFNCYFPLQSYGFQPTNGVGYQPYRVVVLSFELLHAPATVCGPKKST
490 500 510 520 530
NLVKNKCVNFNFNGLTGTGVLTESNKKFLPFQQFGRDIADTTDAVRDPQT
540 550 560 570 580
LEILDITPCSFGGVSVITPGTNTSNQVAVLYQGVNCTEVPVAIHADQLTP
590 600 610 620 630
TWRVYSTGSNVFQTRAGCLIGAEHVNNSYECDIPIGAGICASYQTQTNSP
640 650 660 670 680
SGARSVASQSIIAYTMSLGAENSVAYSNNSIAIPTNFTISVTTEILPVSM
690 700 710 720 730
TKTSVDCTMYICGDSTECSNLLLQYGSFCTQLNRALTGIAVEQDKNTQEV
740 750 760 770 780
FAQVKQIYKTPPIKDFGGFNFSQILPDPSKPSKRSFIEDLLFNKVTLADA
790 800 810 820 830
GFIKQYGDCLGDIAARDLICAQKFNGLTVLPPLLTDEMIAQYTSALLAGT
840 850 860 870 880
ITSGWTFGAGAALQIPFAMQMAYRFNGIGVTQNVLYENQKLIANQFNSAI
890 900 910 920 930
GKIQDSLSSTASALGKLQDVVNQNAQALNTLVKQLSSNFGAISSVLNDIL
940 950 960 970 980
SRLDKVEAEVQIDRLITGRLQSLQTYVTQQLIRAAEIRASANLAATKMSE
990 1000 1010 1020 1030
CVLGQSKRVDFCGKGYHLMSFPQSAPHGVVFLHVTYVPAQEKNFTTAPAI
1040 1050 1060 1070 1080
CHDGKAHFPREGVFVSNGTHWFVTQRNFYEPQIITTDNTFVSGNCDVVIG
1090 1100 1110 1120 1130
IVNNTVYDPLQPELDSFKEELDKYFKNHTSPDVDLGDISGINASVVNIQK
1140 1150 1160 1170 1180
EIDRLNEVAKNLNESLIDLQELGKYEQYIKGSGRENLYFQGGGGSGYIPE
1190 1200 1210 1220 1230
APRDGQAYVRKDGEWVLLSTFLGHHHHHHHH
1240 1250 1260
1a
1b
1c

Supplementary Fig 11. Coverage map of MS/MS peptides detected for SARS-CoV-2 S_{ecto} in isolation (sample 1a), or in the presence of inactive (S441A, sample 1b) and active (S441, sample 1c) TMPRSS2. The sample was digested with GluC as described in the methods section.

S

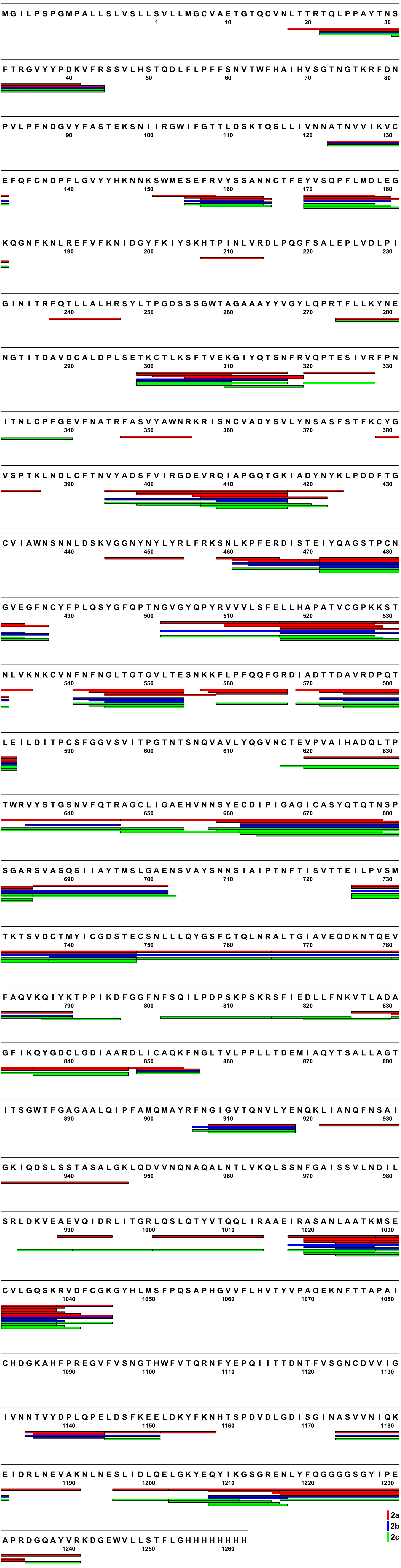
MGILPSPGMPALLSLVSLLSVLLMGCVAETGTQCVNLTTRTQLPPAYTNS
1 10 20 30
FTRGVYYPDKVFRSSVLHSTQDLFLPFFSNVTWFHAIHVSGTNGTKRFDN
40 50 60 70 80
PVLPFNDGVYFASTEKSNIIRGWIFGTTLDSKTQSLLIVNNATNVVIKVC
90 100 110 120 130
EFQFCNDPFLGVYYHKNNKSWMESEFRVYSSANNCTFEYVSQPFLMDLEG
140 150 160 170 180
KQGNFKNLREFVFKNIDGYFKIYSKHTPINLVRDLPQGFSALEPLVDLPI
190 200 210 220 230
GINITRFQTLLALHRSYLTPGDSSSGWTAGAAAYYVGYLQPRTFLLKYNE
240 250 260 270 280
NGTITDAVDCALDPLSETKCTLKSFTVEKGIYQTSNFRVQPTESIVRFPN
290 300 310 320 330
ITNLCPFGEVFNATRFASVYAWNRKRISNCVADYSVLYNSASFSTFKCYG
340 350 360 370 380
VSPTKLNDLCFTNVYADSFVIRGDEVRQIAPGQTGKIADYNYKLPDDFTG
390 400 410 420 430
CVIAWNSNNLDSKVGGNYNYLYRLFRKSNLKPFERDISTEIYQAGSTPCN
440 450 460 470 480
GVEGFNCYFPLQSYGFQPTNGVGYQPYRVVVLSFELLHAPATVCGPKKST
490 500 510 520 530
NLVKNKCVNFNFNGLTGTGVLTESNKKFLPFQQFGRDIADTTDAVRDPQT
540 550 560 570 580
LEILDITPCSFGGVSVITPGTNTSNQVAVLYQGVNCTEVPVAIHADQLTP
590 600 610 620 630
TWRVYSTGSNVFQTRAGCLIGAEHVNNSYECDIPIGAGICASYQTQTNSP
640 650 660 670 680
SGARSVASQSIIAYTMSLGAENSVAYSNNSIAIPTNFTISVTTEILPVSM
690 700 710 720 730
TKTSVDCTMYICGDSTECSNLLLQYGSFCTQLNRALTGIAVEQDKNTQEV
740 750 760 770 780
FAQVKQIYKTPPIKDFGGFNFSQILPDPSKPSKRSFIEDLLFNKVTLADA
790 800 810 820 830
GFIKQYGDCLGDIAARDLICAQKFNGLTVLPPLLTDEMIAQYTSALLAGT
840 850 860 870 880
ITSGWTFGAGAALQIPFAMQMAYRFNGIGVTQNVLYENQKLIANQFNSAI
890 900 910 920 930
GKIQDSLSSTASALGKLQDVVNQNAQALNTLVKQLSSNFGAISSVLNDIL
940 950 960 970 980
SRLDKVEAEVQIDRLITGRLQSLQTYVTQQLIRAAEIRASANLAATKMSE
990 1000 1010 1020 1030
CVLGQSKRVDFCGKGYHLMSFPQSAPHGVVFLHVTYVPAQEKNFTTAPAI
1040 1050 1060 1070 1080
CHDGKAHFPREGVFVSNGTHWFVTQRNFYEPQIITTDNTFVSGNCDVVIG
1090 1100 1110 1120 1130
IVNNTVYDPLQPELDSFKEELDKYFKNHTSPDVDLGDISGINASVVNIQK
1140 1150 1160 1170 1180
EIDRLNEVAKNLNESLIDLQELGKYEQYIKGSGRENLYFQGGGGSGYIPE
1190 1200 1210 1220 1230
APRDGQAYVRKDGEWVLLSTFLGHHHHHHHH
1240 1250 1260
2a
2b
2c

Supplementary Fig 12. Coverage map of MS/MS peptides detected for SARS-CoV-2 S_{ecto} in the presence of ACE2 (sample 2a), ACE2 and inactive TMPRSS2 (S441A, sample 2b) or ACE2 and active TMPRSS2 (S441, sample 2c). The sample was digested with GluC as described in the methods section.

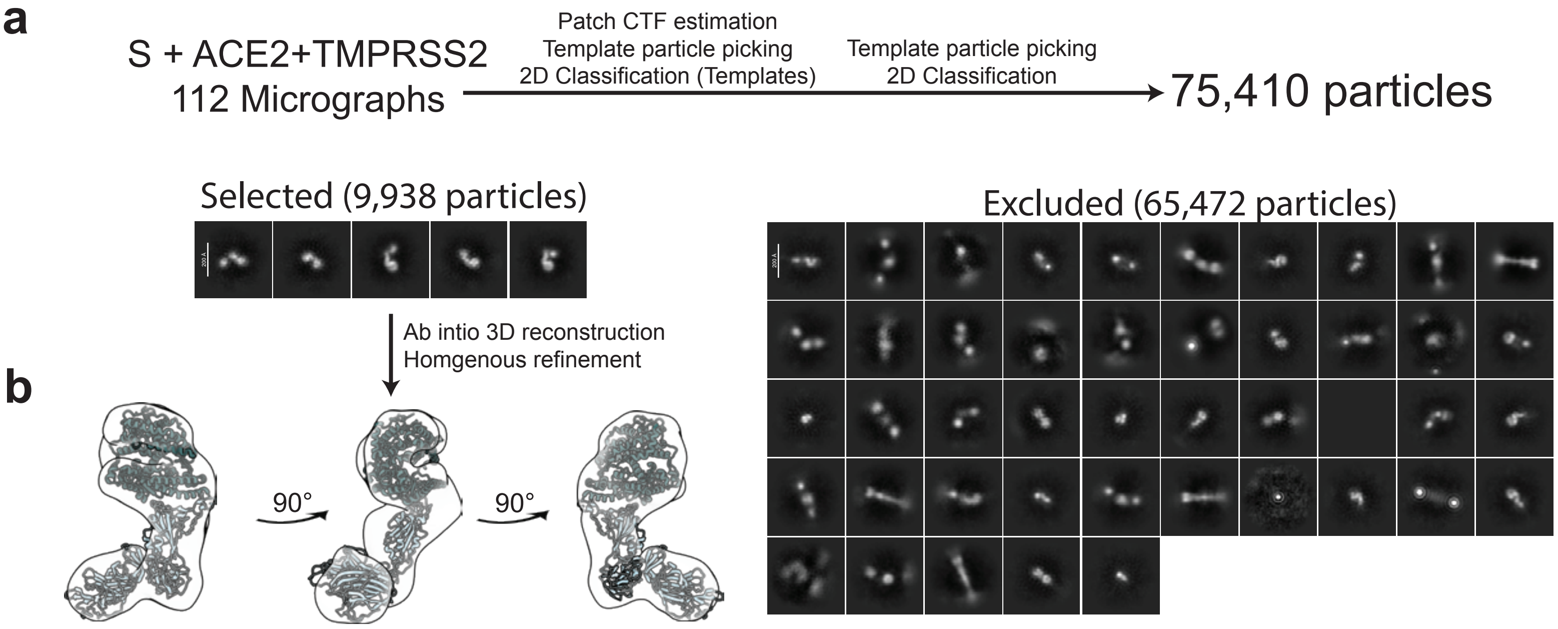

a
S + ACE2+TMPRSS2
112 Micrographs
Patch CTF estimation
Template particle picking
2D Classification (Templates)
Template particle picking
2D Classification
75,410 particles
Selected (9,938 particles)
Excluded (65,472 particles)
Ab intio 3D reconstruction
Homgenous refinement
b
90°
90°

Supplementary Figure 13. EM data processing workflow for shed SARS-CoV-2 S_1 subunit only observed in the presence of ACE2 and active TMPRSS for the first biological replicate. **a,** Early processing steps and 2D classification of particles picked with ACE2-bound S_1 subunit templates obtained from the same dataset as that in Supplementary Figure 9. **b,** 3D reconstruction shown in multiple orientations obtained with the subset of particles selected for the ACE2-bound S_1 subunit shown as semi-transparent grey surface docked with a previously determined structure of ACE2-bound S_1 subunit (PDB 7A92) rendered as ribbons.

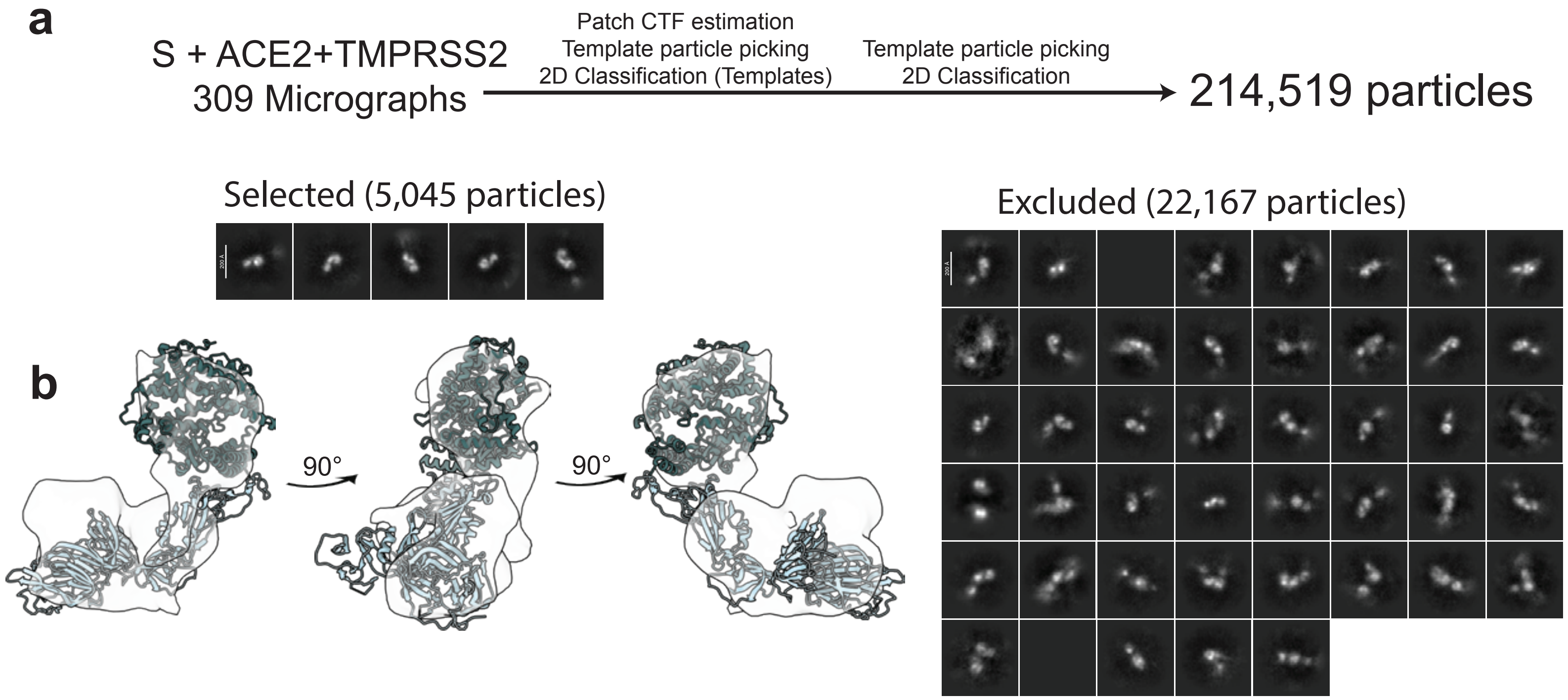
a
S + ACE2+TMPRSS2
309 Micrographs
Patch CTF estimation
Template particle picking
2D Classification (Templates)
Template particle picking
2D Classification
214,519 particles
Selected (5,045 particles)
Excluded (22,167 particles)
b
90°
90°

Supplementary Figure 14. EM data processing workflow of shed SARS-CoV-2 S_1 subunit only observed in the presence of ACE2 and active TMPRSS2 for the second biological replicate. a, Early processing steps and 2D classification of particles picked with ACE2-bound S_1 subunit templates obtained from the same dataset as that in Supplementary Figure 10. **b,** 3D reconstruction shown in multiple orientations obtained with the subset of particles selected for the ACE2-bound S_1 subunit shown as semi-transparent grey surface docked with a previously determined structure of ACE2-bound S_1 subunit (PDB 7A92) rendered as ribbons.

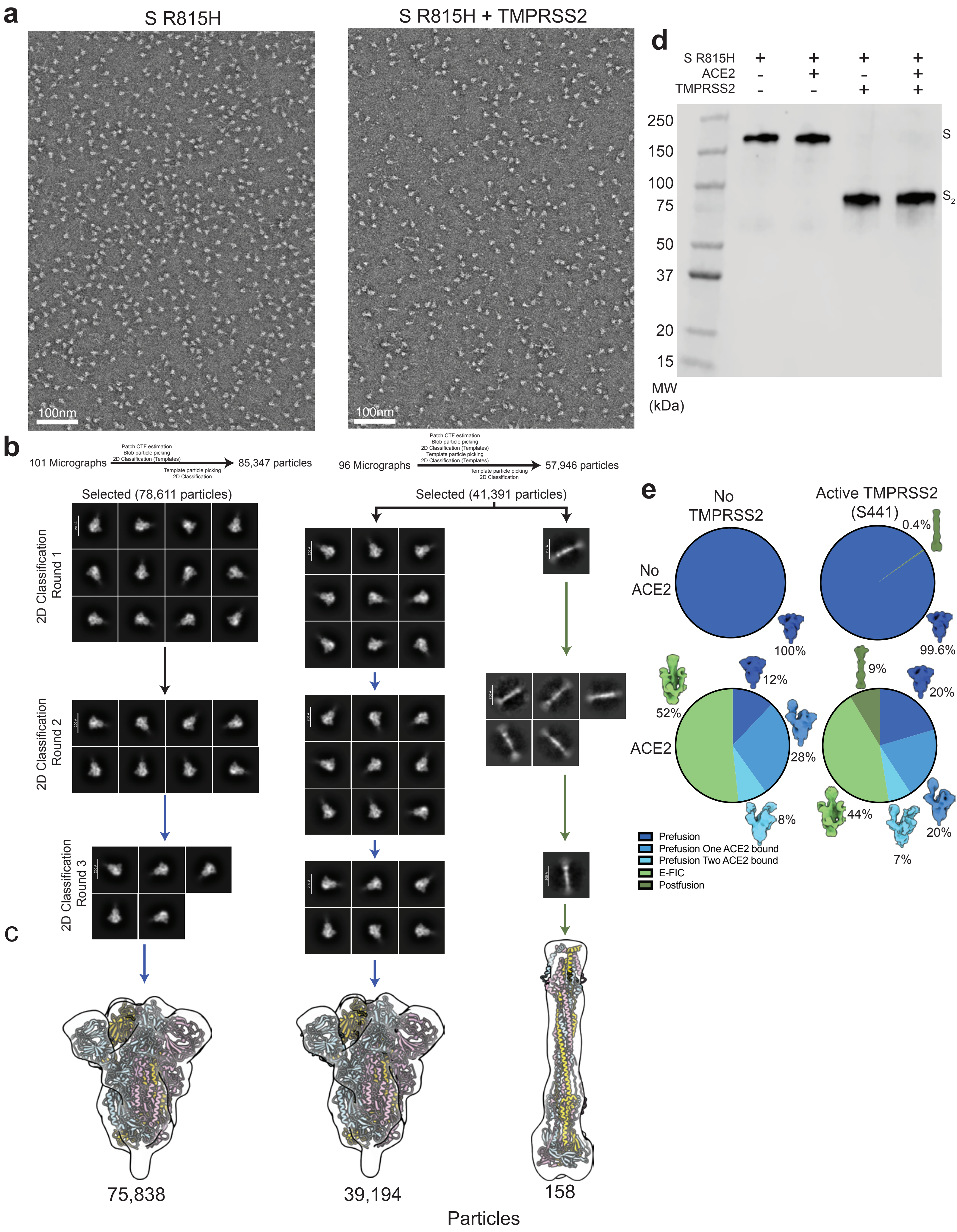
a
S R815H
S R815H + TMPRSS2
100nm
100nm
b
101 Micrographs
Patch CTF estimation
Blob particle picking
2D Classification (Templates)
Template particle picking
2D Classification
85,347 particles
96 Micrographs
57,946 particles
Selected (78,611 particles)
Selected (41,391 particles)
2D Classification Round 1
2D Classification Round 2
2D Classification Round 3
c
75,838
39,194
158
Particles
d
S R815H + + + +
ACE2 - + - +
TMPRSS2 - - + +
250
150
100
75
50
37
20
15
MW (kDa)
S
S_2
e
No TMPRSS2
Active TMPRSS2 (S441)
No ACE2
ACE2
100%
0.4%
99.6%
12%
52%
28%
8%
9%
20%
44%
7%
20%
Prefusion
Prefusion One ACE2 bound
Prefusion Two ACE2 bound
E-FIC
Postfusion

Supplementary Fig 15. EM data processing workflow of SARS-CoV-2 S_{ecto} R815H in the presence or absence of active TMPRSS2 for the first biological replicate. a, Representative electron micrographs for negatively stained apo SARS-CoV-2 S_{ecto} R815H incubated (or not) with 0.2 µM of S441 (active) TMPRSS2 ectodomain for 45 minutes at 4°C in the absence of ACE2. The final SARS-CoV-2 S_{ecto} R815H concentration was 0.65 µM. **b,** Iterative 2D classification of the particle images for the corresponding datasets. **c,** 3D reconstructions obtained with the subset of particles selected for each conformation shown as semi-transparent grey surfaces docked with previously determined structures for prefusion S (PDB 7K43) and postfusion S (PDB 8FDW) rendered as ribbons. **d**, Western blot analysis of SARS-CoV-2 S_{ecto} R815H incubated with ACE2 and/or TMPRSS2 (as described in (a)) detected with the stem helix-directed B6 as primary antibody. **e,** Distribution of conformations detected by single particle EM for negatively stained prefusion SARS-CoV-2 S_{ecto} R815H at a concentration of 0.65 µM incubated (or not) with 2 µM of the monomeric human ACE2 (peptidase) ectodomain for 5 minutes prior to incubation (or not) with the TMPRSS2 ectodomain at a concentration of 0.2 µM for 45 min. All steps were carried out at 4˚C. Pie charts show the distribution of selected particles in each conformation and corresponding 3D reconstructions for the indicated datasets.

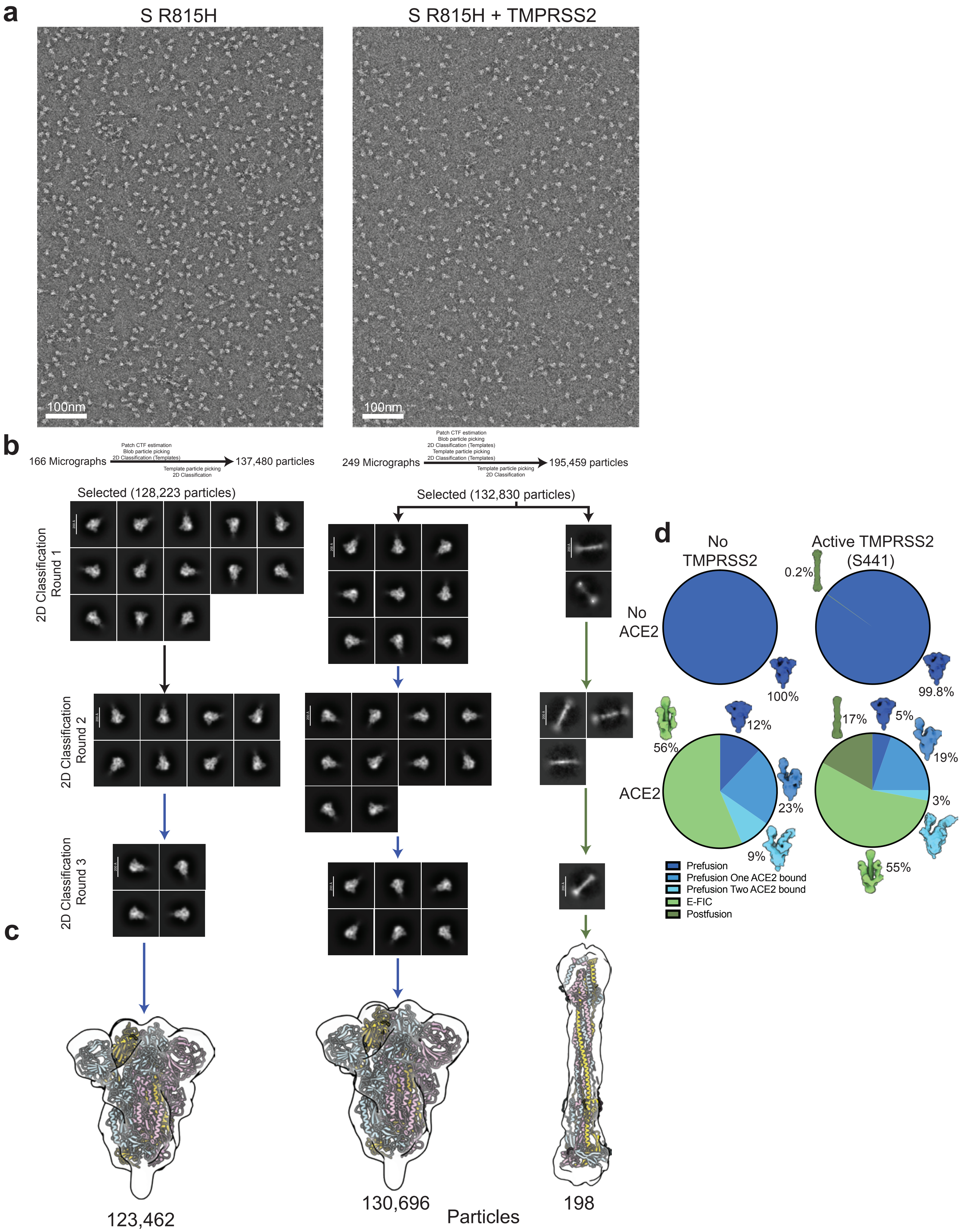

a
S R815H
S R815H + TMPRSS2
100nm
100nm
b
166 Micrographs
Patch CTF estimation
Blob particle picking
2D Classification (Templates)
Template particle picking
2D Classification
137,480 particles
249 Micrographs
Patch CTF estimation
Blob particle picking
2D Classification (Templates)
Template particle picking
2D Classification (Templates)
Template particle picking
2D Classification
195,459 particles
Selected (128,223 particles)
Selected (132,830 particles)
2D Classification Round 1
2D Classification Round 2
2D Classification Round 3
c
123,462
130,696
198
Particles
d
No TMPRSS2
Active TMPRSS2 (S441)
No ACE2
ACE2
100%
0.2%
99.8%
56%
12%
23%
9%
17%
5%
19%
3%
55%
Prefusion
Prefusion One ACE2 bound
Prefusion Two ACE2 bound
E-FIC
Postfusion

Supplementary Fig 16. EM data processing workflow of SARS-CoV-2 S_{ecto} R815H in the presence or absence of active TMPRSS2 for the second biological replicate. a, Representative electron micrographs for negatively stained apo SARS-CoV-2 S_{ecto} R815H incubated (or not) with 0.2 μM of S441 (active) TMPRSS2 ectodomain for 45 minutes at 4°C in the absence of ACE2. The final SARS-CoV-2 S_{ecto} R815H concentration was 0.65 μM. **b,** Iterative 2D classification of the particle images for the corresponding datasets. **c,** 3D reconstructions obtained with the subset of particles selected for each conformation shown as semi-transparent grey surfaces docked with previously determined structures for prefusion S (PDB 7K43) and postfusion S (PDB 8FDW) rendered as ribbons. **d**, Distribution of conformations detected by single particle EM for negatively stained prefusion SARS-CoV-2 S_{ecto} R815H at a concentration of 0.65 μM incubated (or not) with 2 μM of the monomeric human ACE2 (peptidase) ectodomain for 5 minutes prior to incubation (or not) with the TMPRSS2 ectodomain at a concentration of 0.2 μM for 45 min. All steps were carried out at 4˚C. Pie charts show the distribution of selected particles in each conformation and corresponding 3D reconstructions for the indicated datasets.

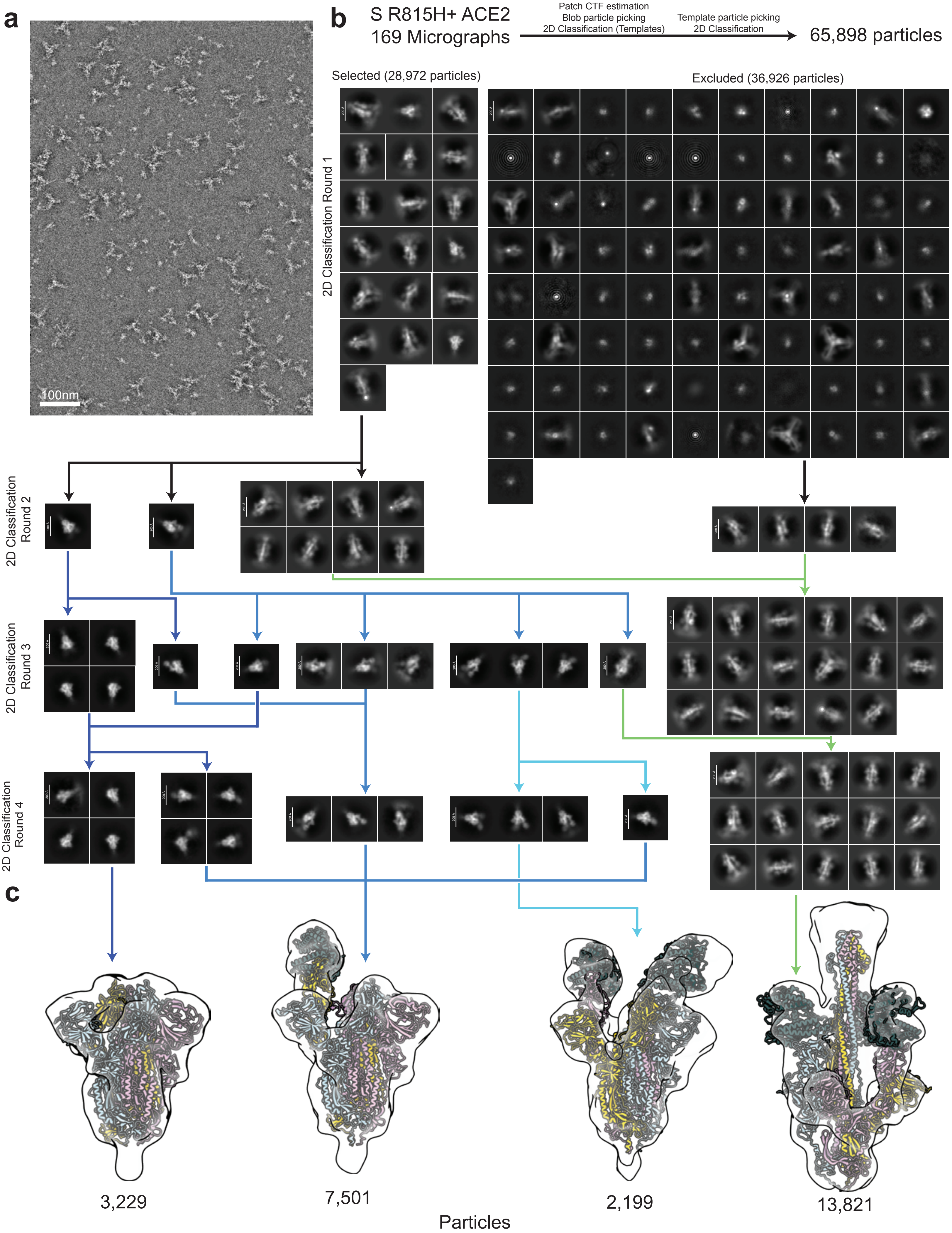
a
100nm
b
S R815H+ ACE2
169 Micrographs
Patch CTF estimation
Blob particle picking
2D Classification (Templates)
Template particle picking
2D Classification
65,898 particles
Selected (28,972 particles)
Excluded (36,926 particles)
2D Classification Round 1
2D Classification Round 2
2D Classification Round 3
2D Classification Round 4
c
3,229
7,501
2,199
13,821
Particles

Supplementary Fig 17. EM data processing workflow of SARS-CoV-2 S_{ecto} R815H in the presence of ACE2 for the first biological replicate. a, Representative electron micrograph for negatively stained SARS-CoV-2 S_{ecto} R815H at 0.65 µM incubated with the monomeric ACE2 (peptidase) ectodomain at a 1:3 molar ratio for 45 minutes at 4˚C. **b,** Iterative 2D classification of the particle images leading to identification of distinct S conformations. **c,** 3D reconstructions obtained with the subset of particles selected for each conformation shown as semi-transparent grey surfaces docked with previously determined structures for prefusion S (PDB 7K43), one ACE2-bound prefusion S (PDB 7A94), two ACE2-bound prefusion S with one RBD-ACE2 region fit independently (PDB 7A97), and E-FIC (PDB 8Z7P) rendered as ribbons.

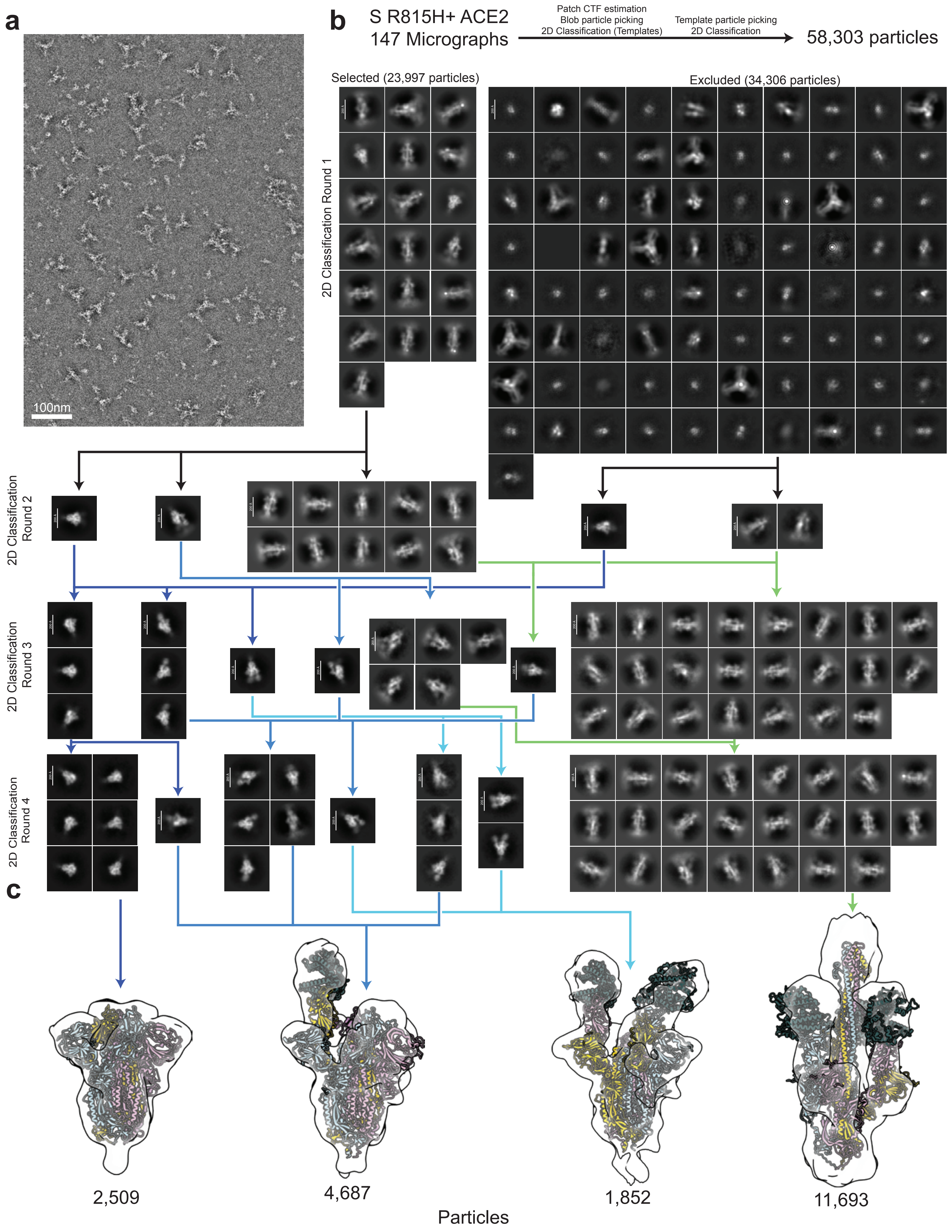
a
100nm
b
S R815H+ ACE2
147 Micrographs
Patch CTF estimation
Blob particle picking
2D Classification (Templates)
Template particle picking
2D Classification
58,303 particles
Selected (23,997 particles)
Excluded (34,306 particles)
2D Classification Round 1
2D Classification Round 2
2D Classification Round 3
2D Classification Round 4
c
2,509
4,687
1,852
11,693
Particles

Supplementary Figure 18. EM data processing workflow of SARS-CoV-2 S_{ecto} R815H in the presence of ACE2 for the second biological replicate. a, Representative electron micrograph for negatively stained SARS-CoV-2 S_{ecto} R815H at 0.65 µM incubated with the monomeric ACE2 (peptidase) ectodomain at a 1:3 molar ratio for 45 minutes at 4˚C. **b,** Iterative 2D classification of the particle images leading to identification of distinct S conformations. **c,** 3D reconstructions obtained with the subset of particles selected for each conformation shown as semi-transparent grey surfaces docked with previously determined structures for prefusion S (PDB 7K43), one ACE2-bound prefusion S (PDB 7A94), two ACE2-bound prefusion S with one RBD-ACE2 region fit independently (PDB 7A97), and E-FIC (PDB 8Z7P) rendered as ribbons.

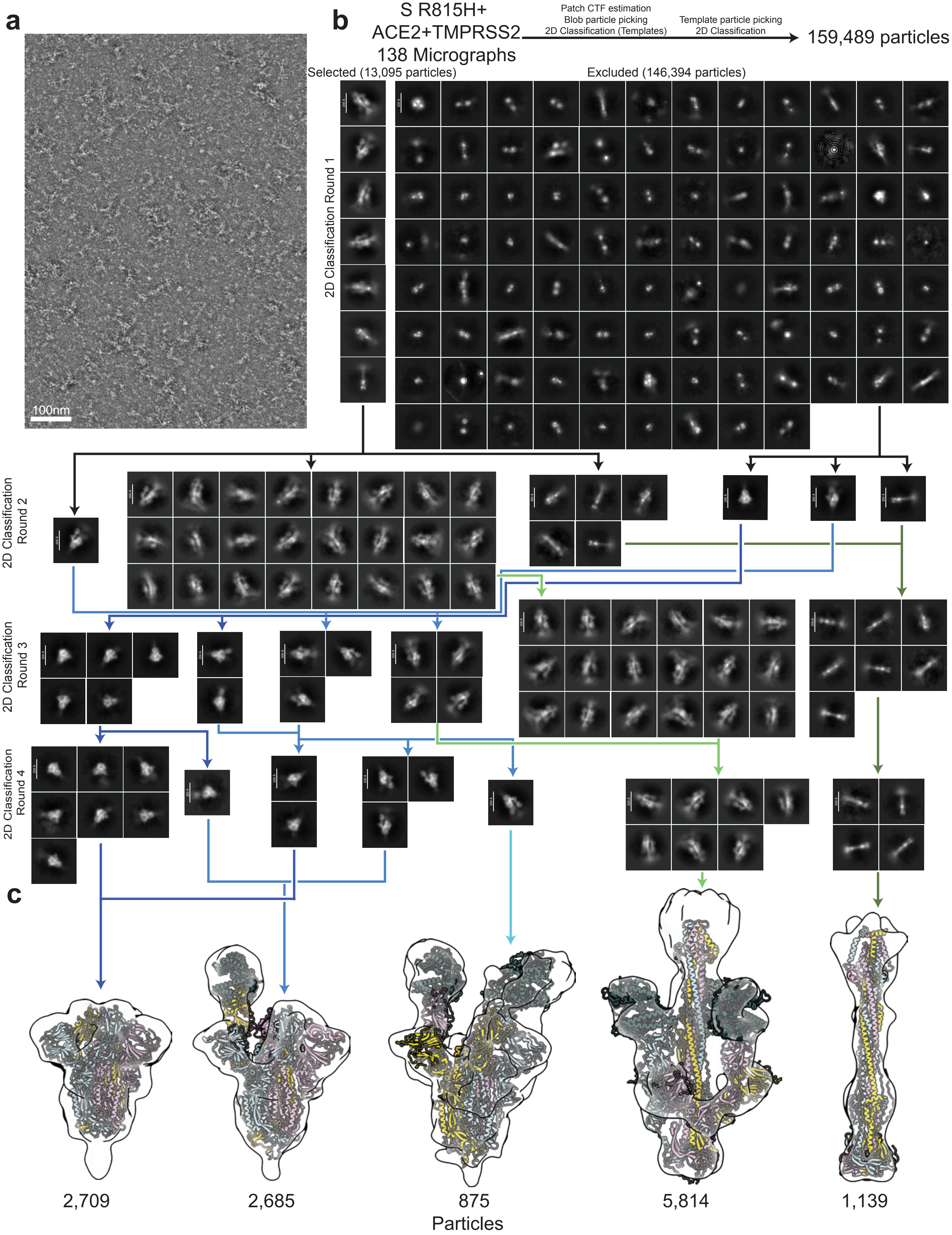

a
100nm
b
S R815H+
ACE2+TMPRSS2
138 Micrographs
Patch CTF estimation
Blob particle picking
2D Classification (Templates)
Template particle picking
2D Classification
159,489 particles
Selected (13,095 particles)
Excluded (146,394 particles)
2D Classification Round 1
2D Classification Round 2
2D Classification Round 3
2D Classification Round 4
c
2,709
2,685
875
5,814
1,139
Particles

Supplementary Figure 19. EM data processing workflow of SARS-CoV-2 S_{ecto} R815H in the presence of ACE2 and active TMPRSS2 for the first biological replicate. a, Representative electron micrograph for negatively stained SARS-CoV-2 S_{ecto} R815H at 0.65 μM incubated with the monomeric human ACE2 (peptidase) ectodomain at a 1:3 molar ratio for 5 min followed by 0.2 μM of the S441 TMPRSS2 ectodomain for 45 minutes. All steps were carried out at 4˚C. **b,** Iterative 2D classification of the particle images leading to identification of distinct S conformations. **c,** 3D reconstructions obtained with the subset of particles selected for each conformation shown as semi-transparent grey surfaces docked with previously determined structures of prefusion S (PDB 7K43), one ACE2-bound prefusion S (PDB 7A94), two ACE2-bound prefusion S (PDB 7A97), E-FIC (PDB 8Z7P), and postfusion S (PDB 8FDW) rendered as ribbons.

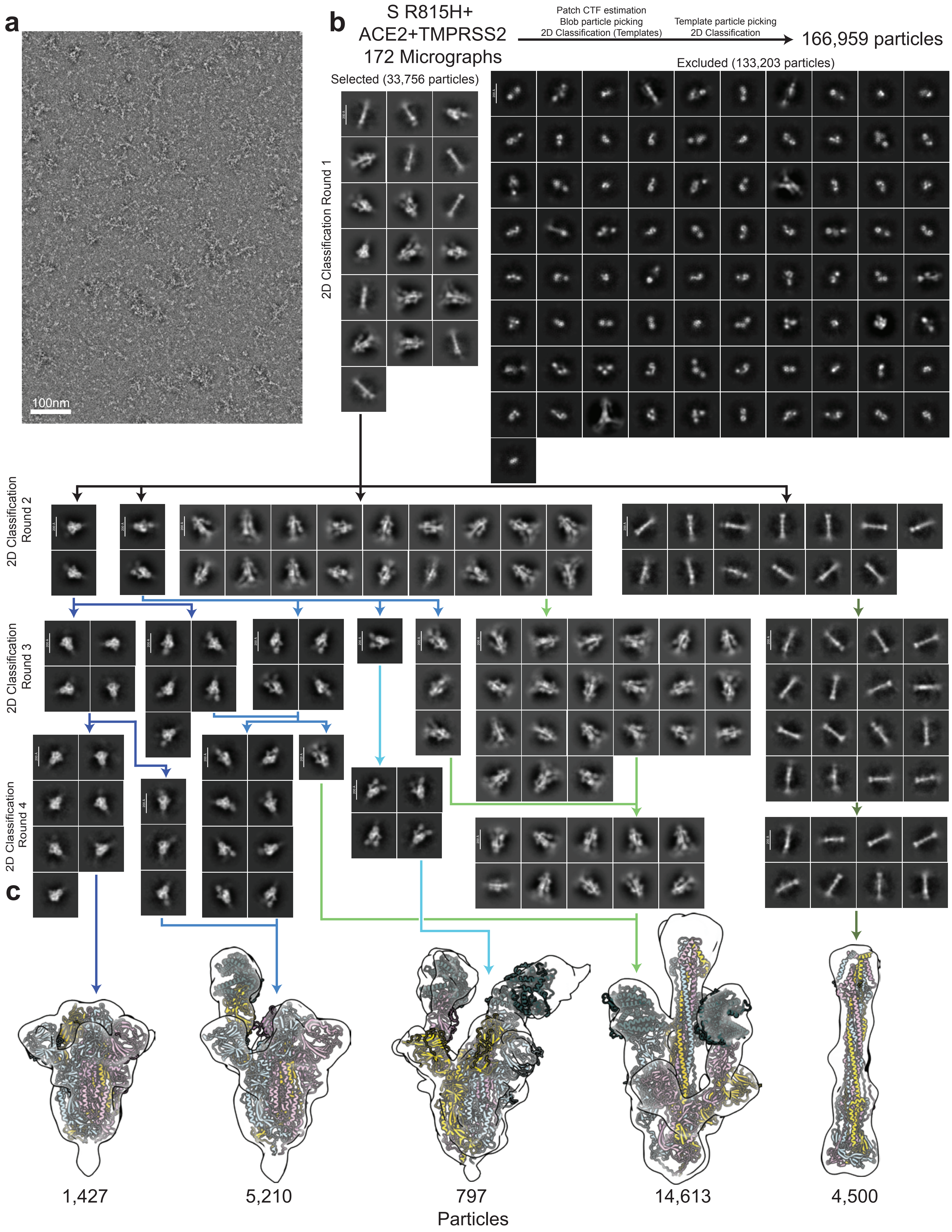
a
100nm
b
S R815H+
ACE2+TMPRSS2
172 Micrographs
Patch CTF estimation
Blob particle picking
2D Classification (Templates)
Template particle picking
2D Classification
166,959 particles
Selected (33,756 particles)
Excluded (133,203 particles)
2D Classification Round 1
2D Classification Round 2
2D Classification Round 3
2D Classification Round 4
c
1,427
5,210
797
14,613
4,500
Particles

Supplementary Fig 20. EM data processing workflow of SARS-CoV-2 S_{ecto} R815H in the presence of ACE2 and active TMPRSS2 for the second biological replicate. a, Representative electron micrograph for negatively stained SARS-CoV-2 S_{ecto} R815H at 0.65 μM incubated with the monomeric human ACE2 (peptidase) ectodomain at a 1:3 molar ratio for 5 min followed by 0.2 μM of the S441 TMPRSS2 ectodomain for 45 minutes. All steps were carried out at 4˚C. **b,** Iterative 2D classification of the particle images leading to identification of distinct S conformations. **c,** 3D reconstructions obtained with the subset of particles selected for each conformation shown as semi-transparent grey surfaces docked with previously determined structures of prefusion S (PDB 7K43), one ACE2-bound prefusion S (PDB 7A94), two ACE2-bound prefusion S (PDB 7A97), E-FIC (PDB 8Z7P), and postfusion S (PDB 8FDW) rendered as ribbons.

Supplementary Table 1. Sequences of the coronavirus S_2' peptides used for biolayer interferometry experiments. All peptides were modified with a C-terminal PEG6-Lys-biotin.

SARS-CoV-2	KPSKRSFIEDLLFNK
HCoV-HKU1	GSSSRSLLEDLLFNK
HCoV-OC43	KASSRSAIEDLLFDK
HCoV-NL63	RIAGRSALEDLLFSK
HCoV-229E	RVAGRSAIEDILFSK
PDCoV	RLGGRSAIEDLLFNK
IBV	SPRRRSFIEDLLFTS
SARS-CoV-1	KPTKRSFIEDLLFNK
MERS-CoV	SRSARSAIEDLLFDK
PDF2180	SSNARSALEELLFDK
CCoV-HuPn-2018	KRKYRSAIEDLLFDK

Supplementary Table 2. Biolayer interferometry kinetics and affinity of binding of the S441A hTMPRSS2 ectodomain to the HCoV-NL63 S_2' peptide and HCoV-NL63-S_2'/HKU1-RBD immobilized at the surface of SA biosensors. Data from two biological replicates using independently produced batches of proteins are shown.

	Biotinylated HCoV-NL63 S_2' Peptide		Biotinylated NL63-S_2'/HKU1-RBD Replicate 1		Biotinylated NL63-S_2'/HKU1-RBD Replicate 2	
	**S441A hTMPRSS2 Replicate 1**	**S441A hTMPRSS2 Replicate 2**	**S441A hTMPRSS2 Replicate 1**	**S441A hTMPRSS2 Replicate 2**	**S441A hTMPRSS2 Replicate 1**	**S441A hTMPRSS2 Replicate 2**
**K_D**	6.01 10^{-7} M	4.09 10^{-7} M	2.07 10^{-10} M	2.08 10^{-10} M	1.78 10^{-10} M	2.40 10^{-10} M
**k_{on}**	1.31 10^{5} $M^{-1}s^{-1}$	2.92 10^{5} $M^{-1}s^{-1}$	1.99 10^{6} $M^{-1}s^{-1}$	1.48 10^{6} $M^{-1}s^{-1}$	1.86 10^{6} $M^{-1}s^{-1}$	1.36 10^{6} $M^{-1}s^{-1}$
**k_{off}**	7.86 10^{-2} s^{-1}	1.19 10^{-1} s^{-1}	4.13 10^{-4} s^{-1}	3.09 10^{-4} s^{-1}	3.32 10^{-4} s^{-1}	3.28 10^{-4} s^{-1}

Supplementary Table 3. Biolayer interferometry kinetics and affinity of binding of H1H7 IgG and Fab to S441A hTMPRSS2. Data from one biological replicate with two technical replicates are shown.

	mAb<br>Replicate 1	mAb<br>Replicate 2	Fab<br>Replicate 1	Fab<br>Replicate 2
K_D	$<1.00 \ \ 10^{-12}$ M	$<1.00 \ \ 10^{-12}$ M	$8.36 \ \ 10^{-10}$ M	$4.93 \ \ 10^{-10}$ M
k_{on}	$5.38 \ \ 10^{5}$ $M^{-1}s^{-1}$	$4.80 \ \ 10^{5}$ $M^{-1}s^{-1}$	$2.56 \ \ 10^{5}$ $M^{-1}s^{-1}$	$2.76 \ \ 10^{5}$ $M^{-1}s^{-1}$
k_{off}	$5.06 \ \ 10^{-7}$ s^{-1}	$1.00 \ \ 10^{-7}$ s^{-1}	$2.14 \ \ 10^{-4}$ s^{-1}	$1.36 \ \ 10^{-4}$ s^{-1}

Raw Image

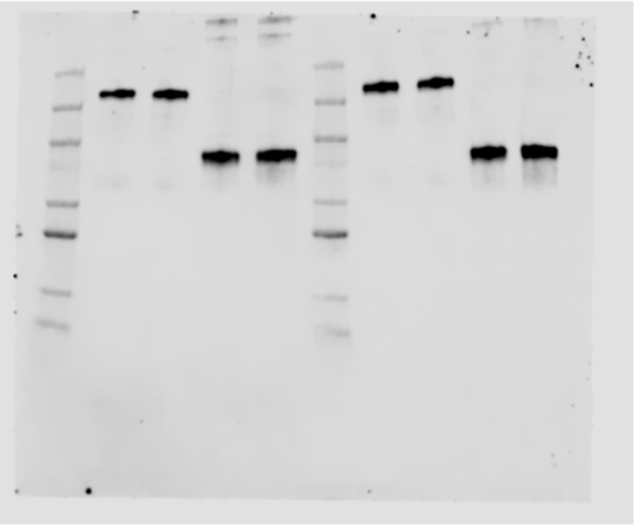

Supplementary Figure 15d

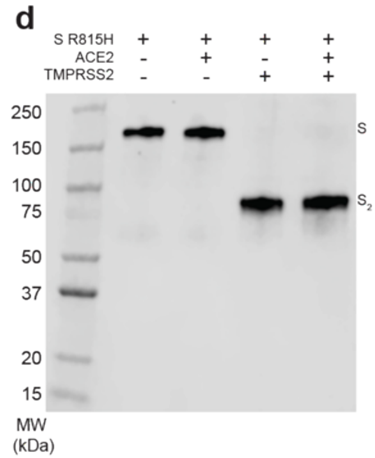

Raw Image

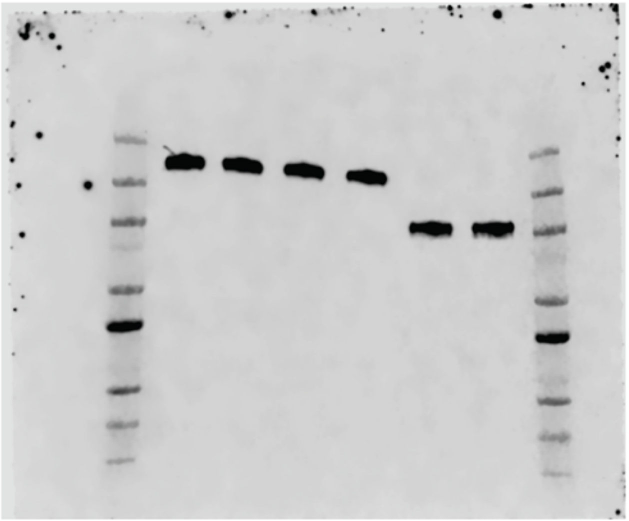

Supplementary Figure 1a

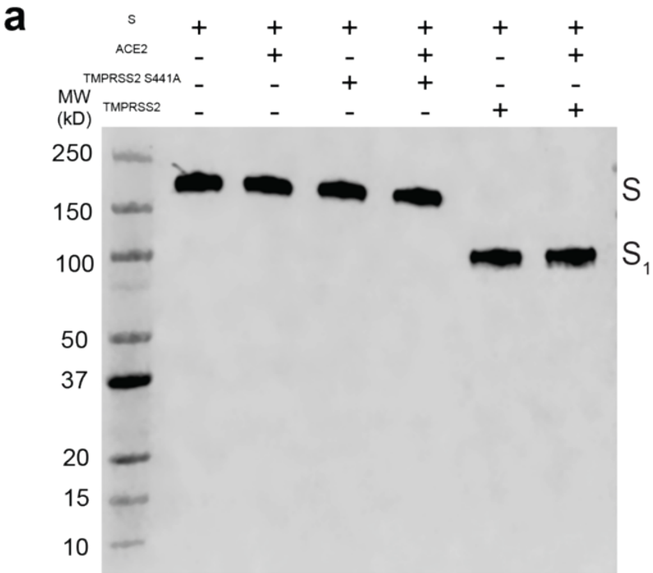

Raw Image

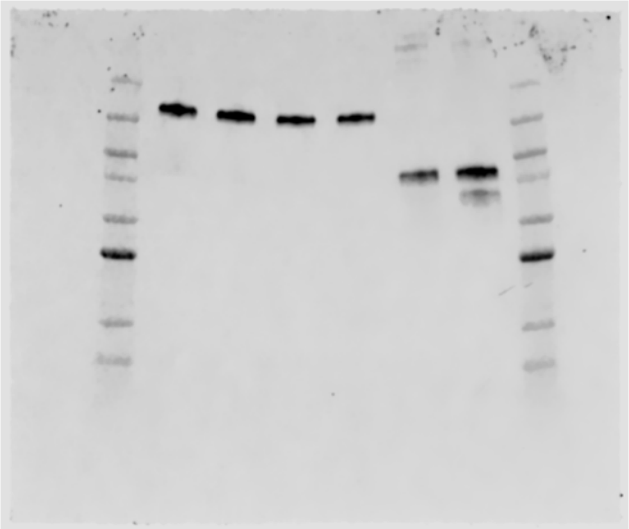

Figure 1c

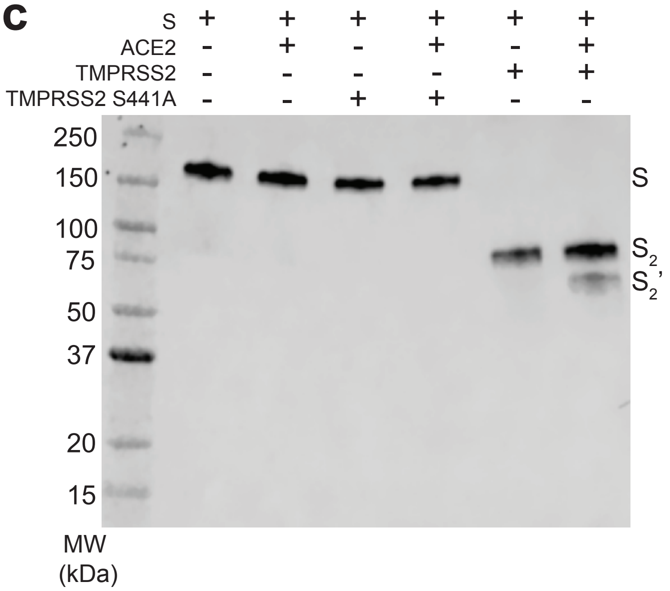

Raw Image

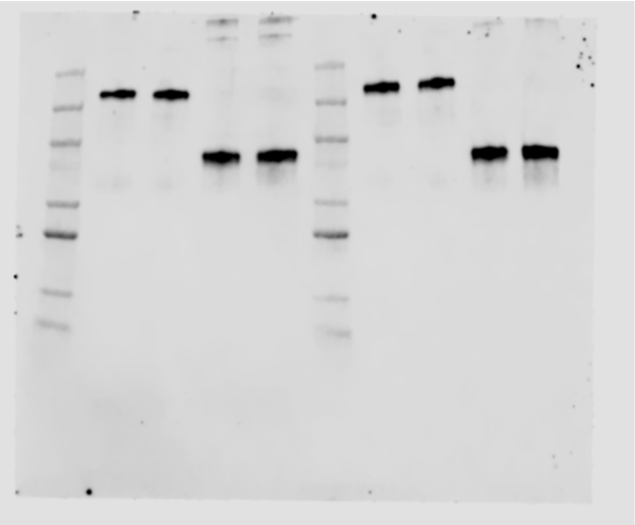

Figure 1g

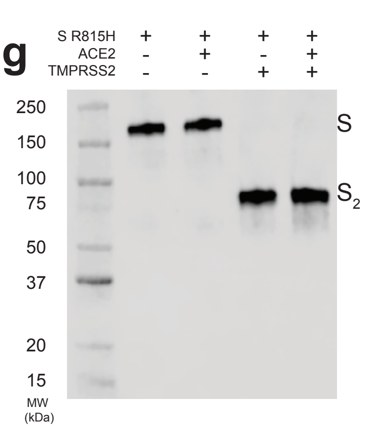

Supplementary Figure 15d

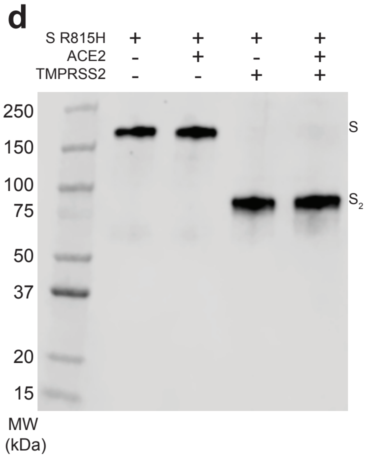

Raw Image

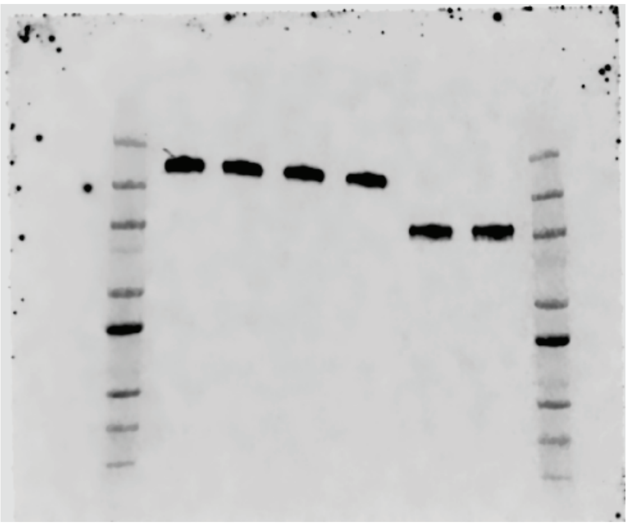

Supplementary Figure 1a

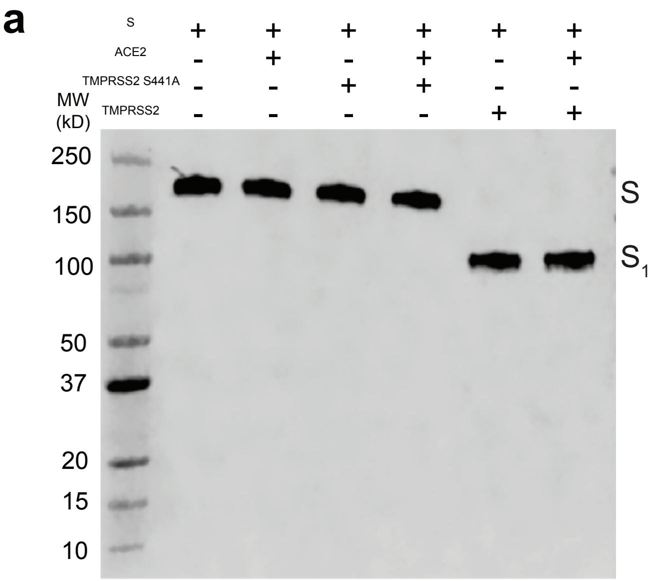

Raw Image

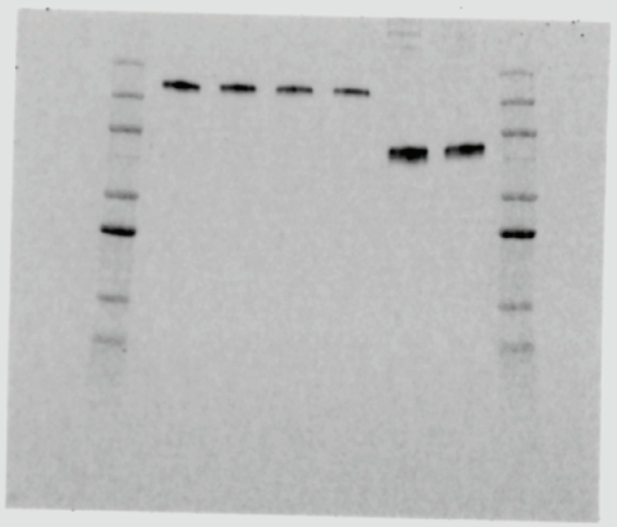

Supplementary Figure 1b

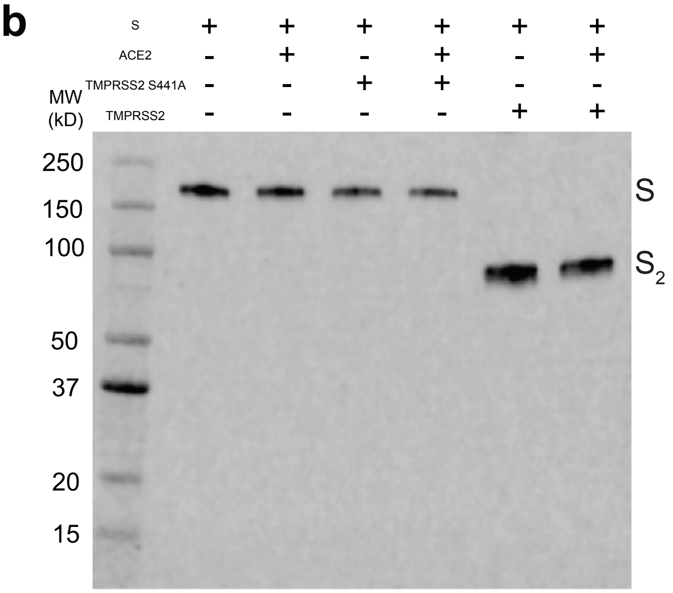

Raw Image

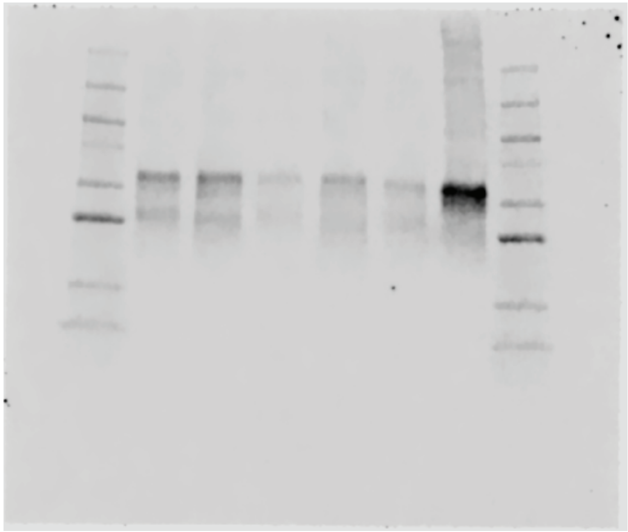

Supplementary Figure 1c

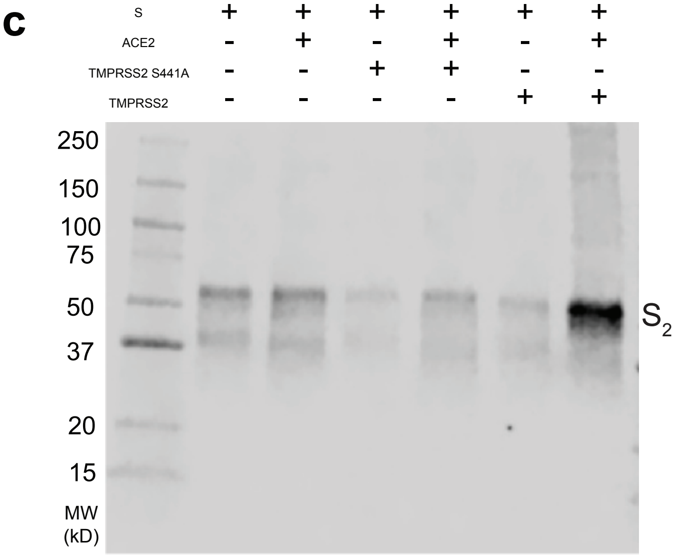

Raw Image

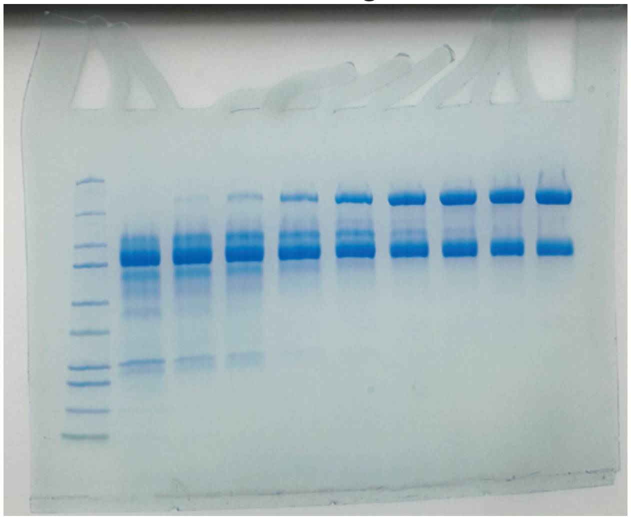

Supplementary Figure 1d

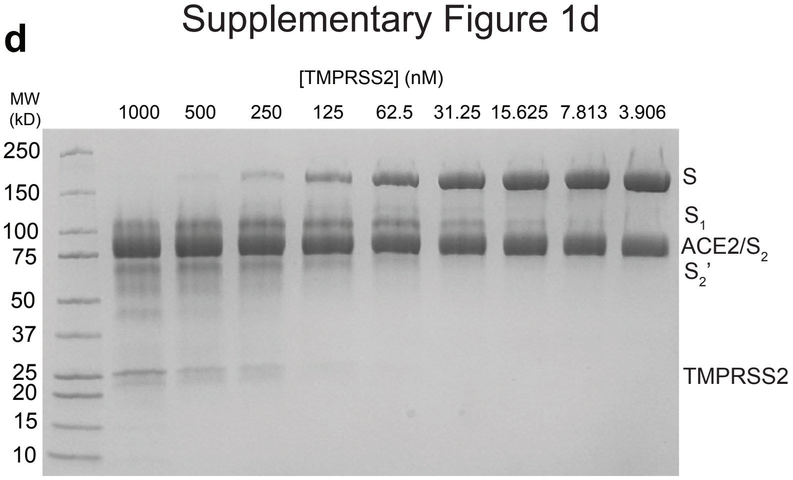

Raw Images

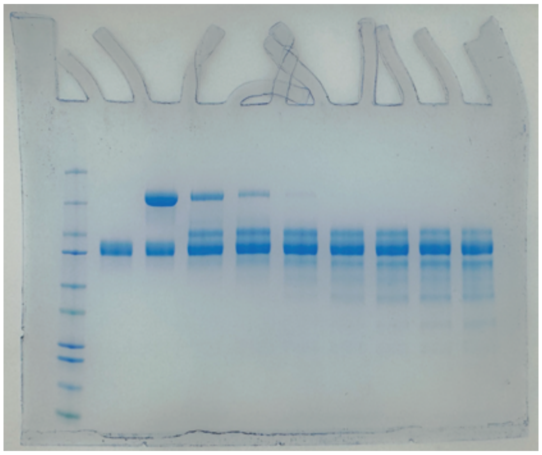

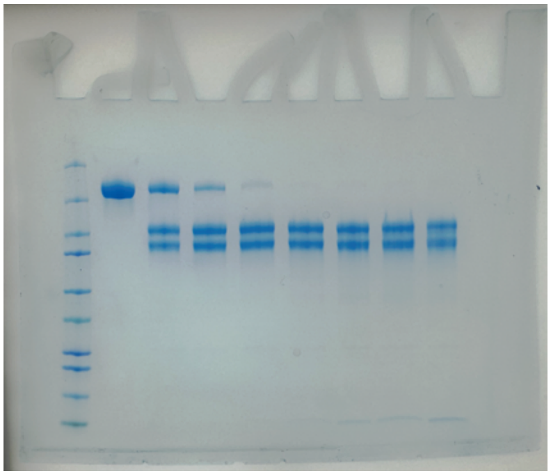

Supplementary Figure 1e

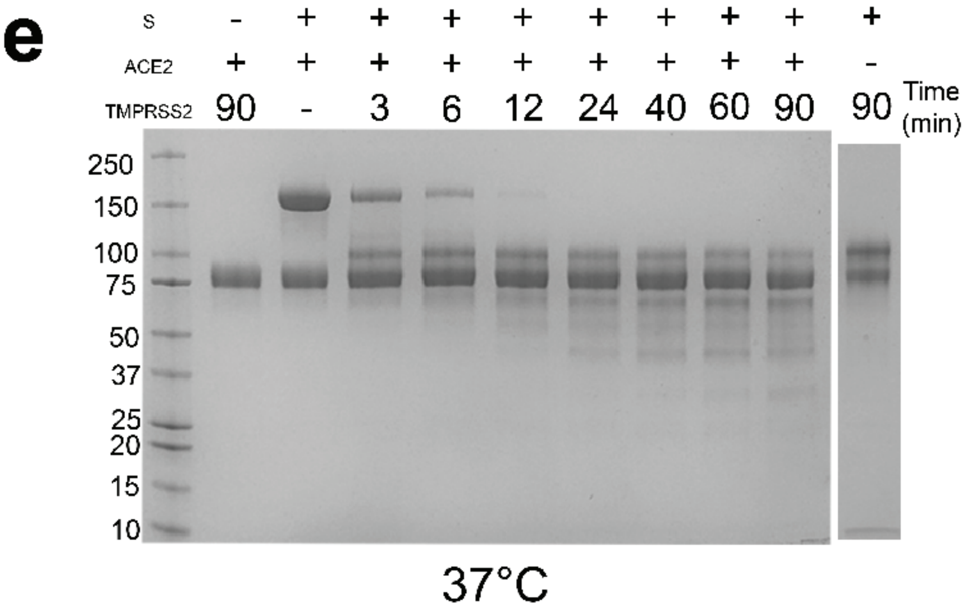

Raw Images

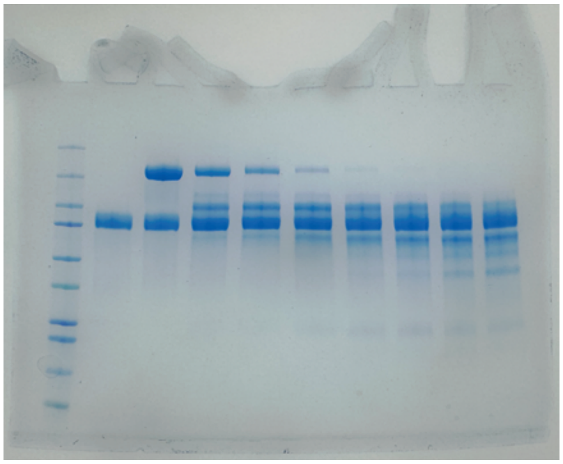

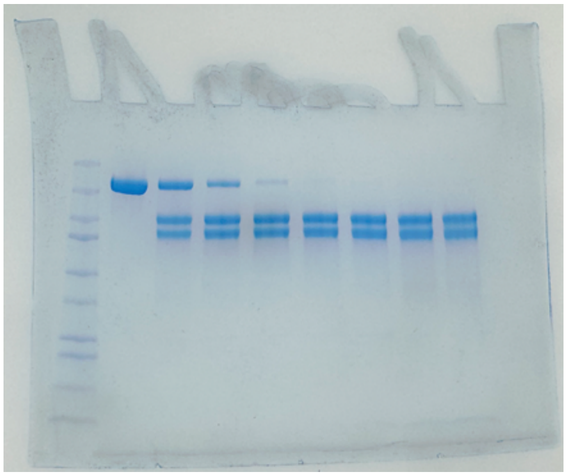

Supplementary Figure 1f

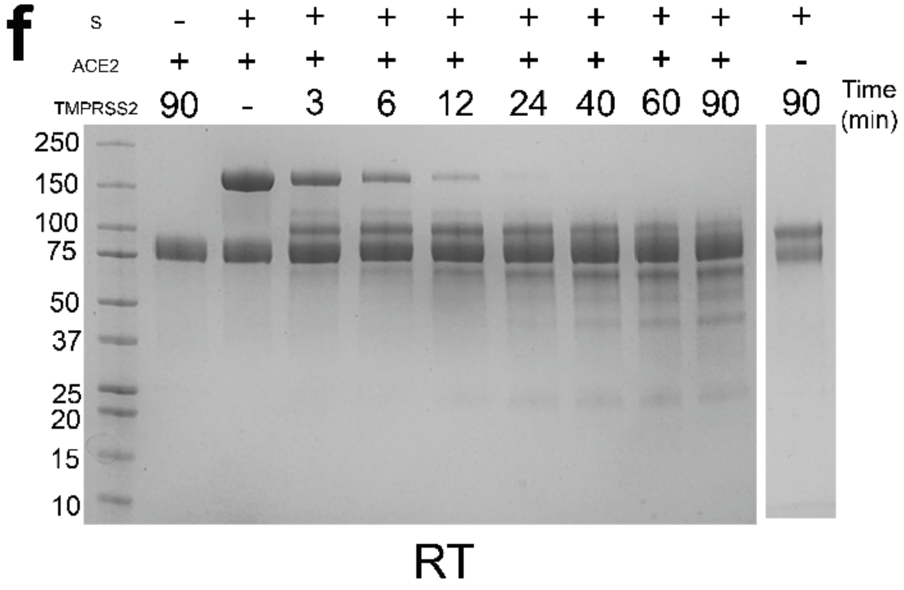

Raw Images

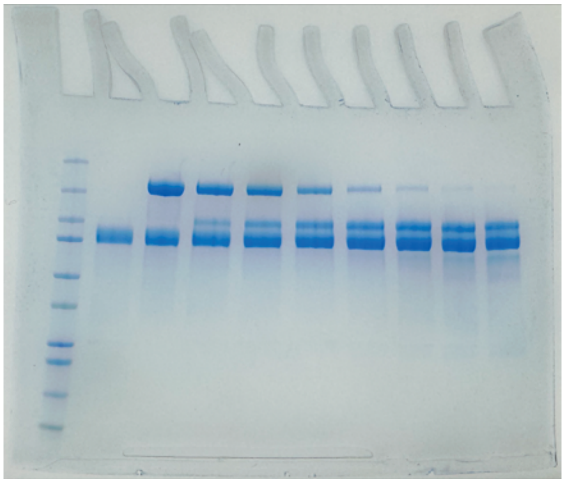

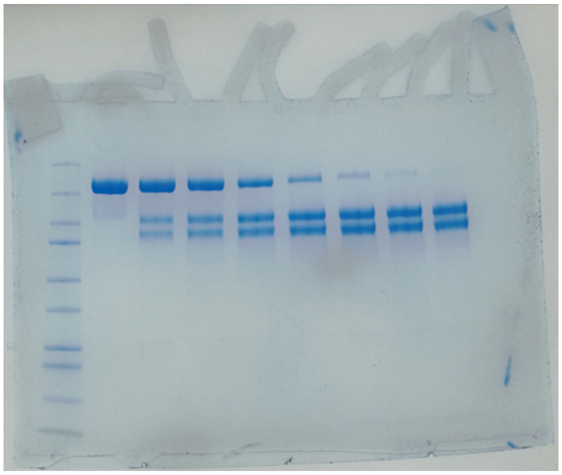

Supplementary Figure 1g

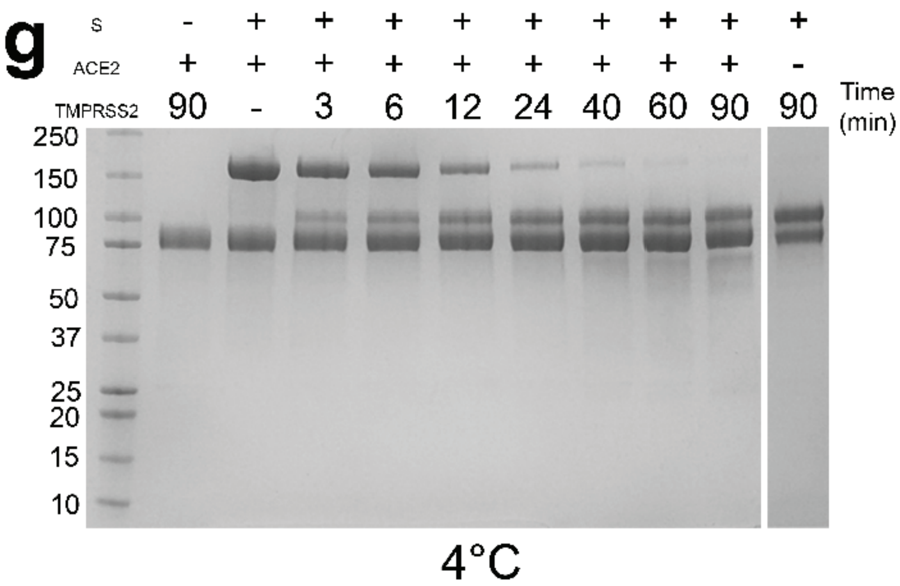

Raw Image

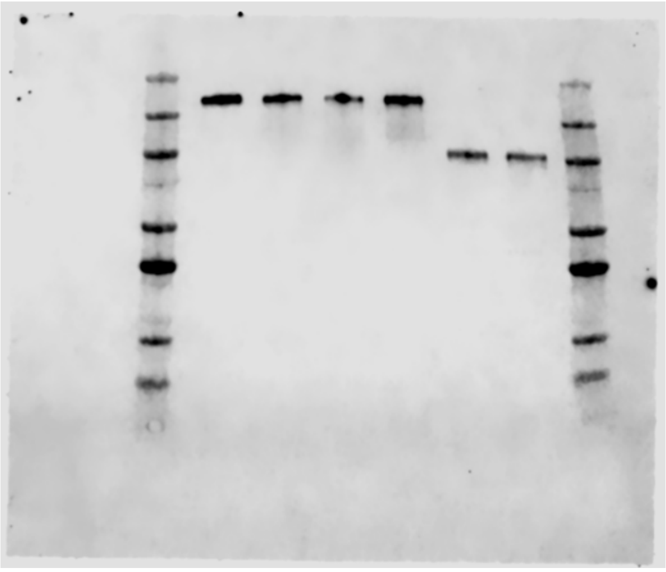

Supplementary Figure 2a

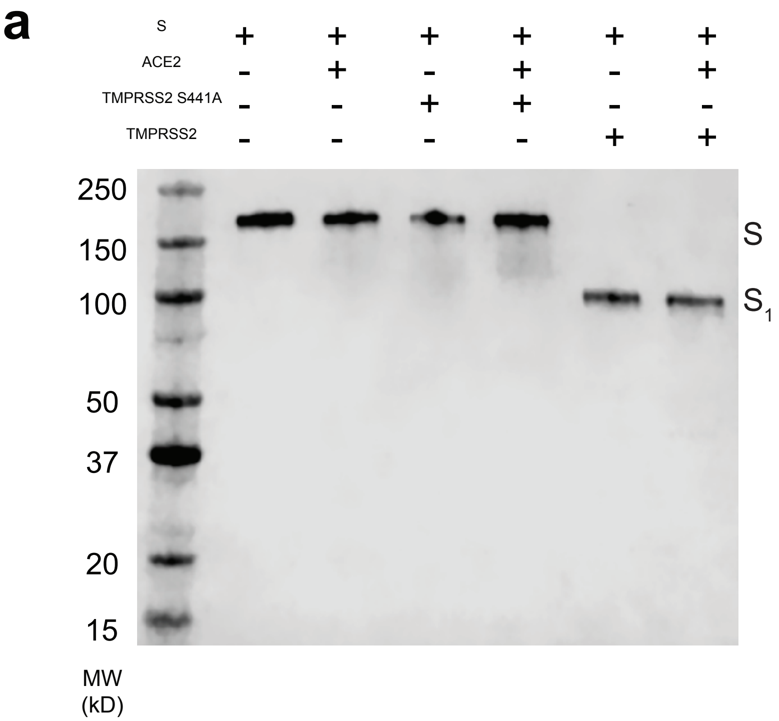

Raw Image

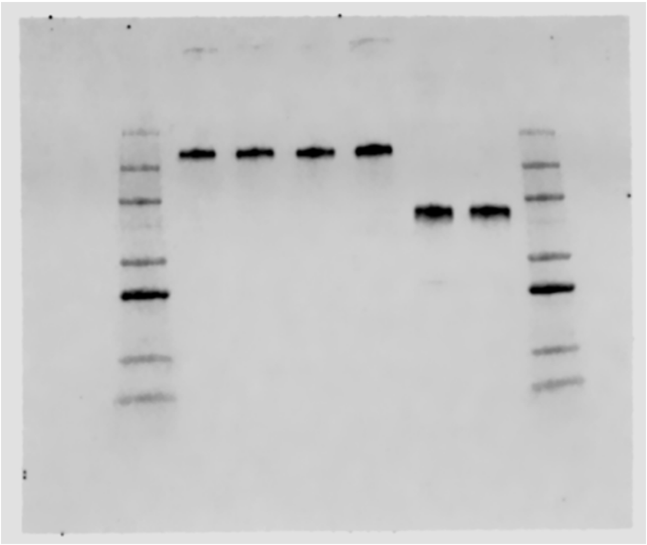

Supplementary Figure 2b

b

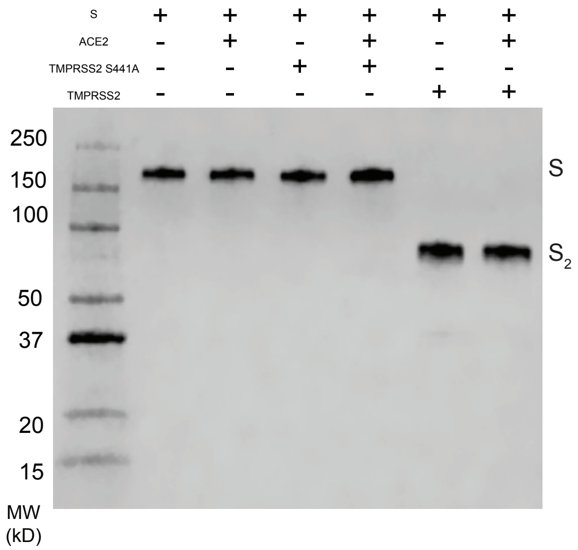

Raw Image

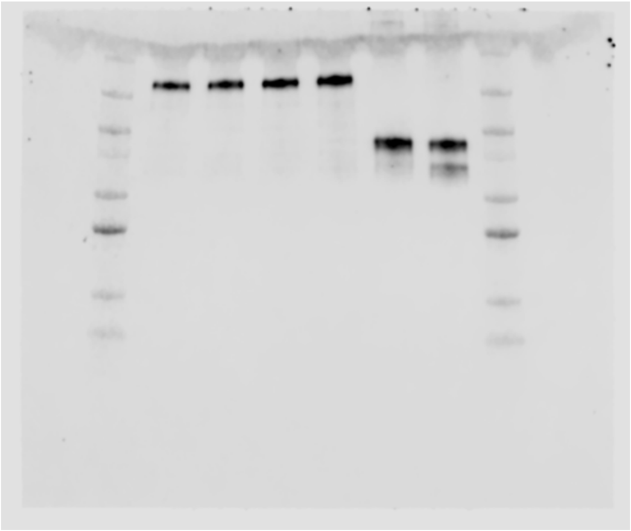

Supplementary Figure 2c

c

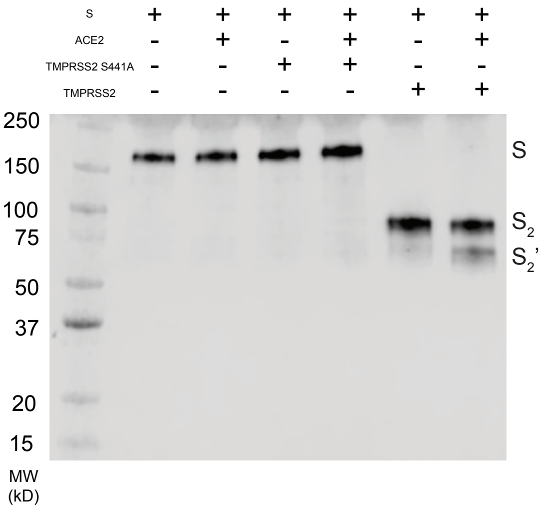

Raw Image

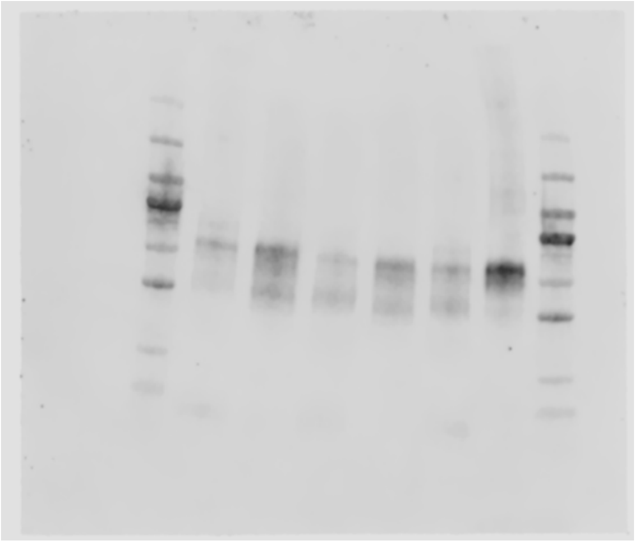

Supplementary Figure 2d

d

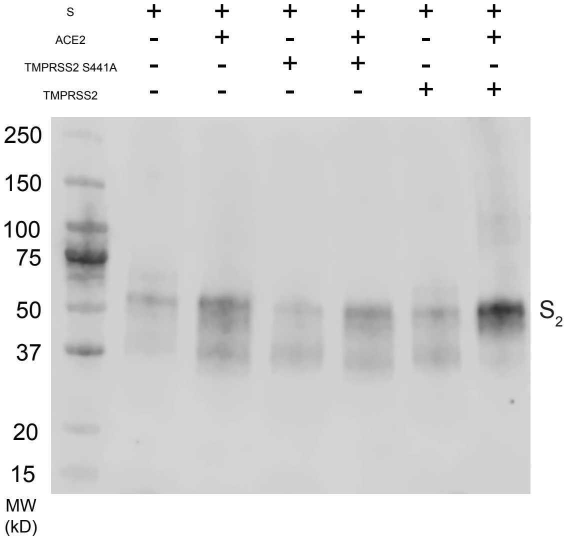

Supplement: Supplementary file 1 — Supplementary Figs. 1–20, Tables 1–3 and Source data for Supplementary Figs. 1, 2 and 15. [file 41594_2026_1801_MOESM1_ESM.pdf]
