# Supplementary material for: TMPRSS2-mediated coronavirus spike activation and inhibition
Source: Nat Struct Mol Biol. 2026 Apr 28;33(5):810–23. doi: 10.1038/s41594-026-01801-y (PMC13186702; doi:10.1038/s41594-026-01801-y)
Supplement: Supplementary file 2 — Reporting Summary [file 41594_2026_1801_MOESM2_ESM.pdf]

nature portfolio

Corresponding author(s): David Veesler

Last updated by author(s): Mar 12, 2026

Reporting Summary

Nature Portfolio wishes to improve the reproducibility of the work that we publish. This form provides structure for consistency and transparency in reporting. For further information on Nature Portfolio policies, see our Editorial Policies and the Editorial Policy Checklist.

Statistics

For all statistical analyses, confirm that the following items are present in the figure legend, table legend, main text, or Methods section.

n/a	Confirmed	
☐	☒	The exact sample size (*n*) for each experimental group/condition, given as a discrete number and unit of measurement
☐	☒	A statement on whether measurements were taken from distinct samples or whether the same sample was measured repeatedly
☐	☒	The statistical test(s) used AND whether they are one- or two-sided<br>*Only common tests should be described solely by name; describe more complex techniques in the Methods section.*
☒	☐	A description of all covariates tested
☐	☒	A description of any assumptions or corrections, such as tests of normality and adjustment for multiple comparisons
☐	☒	A full description of the statistical parameters including central tendency (e.g. means) or other basic estimates (e.g. regression coefficient) AND variation (e.g. standard deviation) or associated estimates of uncertainty (e.g. confidence intervals)
☐	☒	For null hypothesis testing, the test statistic (e.g. *F*, *t*, *r*) with confidence intervals, effect sizes, degrees of freedom and *P* value noted<br>*Give P values as exact values whenever suitable.*
☒	☐	For Bayesian analysis, information on the choice of priors and Markov chain Monte Carlo settings
☒	☐	For hierarchical and complex designs, identification of the appropriate level for tests and full reporting of outcomes
☒	☐	Estimates of effect sizes (e.g. Cohen's *d*, Pearson's *r*), indicating how they were calculated

Our web collection on statistics for biologists contains articles on many of the points above.

Software and code

Policy information about availability of computer code

Data collection	SerialEM 4.2, BioTek Gen5
Data analysis	CryoSPARC 4.6, RELION 3.0 and 5.0, Leginon 3.4, Warp v2.0, Topaz v0.2.5a (implemented in cryoSPARC), ChimeraX 1.10, Coot v0.9.8.93, ISOLDE v1.10.1 (implemented in ChimeraX), Rosetta, Phenix v1.21rc1-5109, GraphPad Prism 10, Sartorius Analysis Software v11.1

For manuscripts utilizing custom algorithms or software that are central to the research but not yet described in published literature, software must be made available to editors and reviewers. We strongly encourage code deposition in a community repository (e.g. GitHub). See the Nature Portfolio guidelines for submitting code & software for further information.

Data

Policy information about availability of data

All manuscripts must include a data availability statement. This statement should provide the following information, where applicable:

- Accession codes, unique identifiers, or web links for publicly available datasets
- A description of any restrictions on data availability
- For clinical datasets or third party data, please ensure that the statement adheres to our policy

The cryoEM maps and atomic models were deposited to the Electron Microscopy Data Bank and Protein Data Bank with accession IDs EMD-70721 and PDB 9OPQ (NL63-S2′/HKU1-RBD + TMPRSS2-S441A), EMD-73786 and PDB 9Z3J (NL63-S2′ + TMPRSS2-S441A +H1H7 Fab + anti-kappa-nanobody), EMD-70722 and PDB 9OPR (H1H7 Fab + anti-kappa-nanobody + TMPRSS2-S441A), EMD-75233 (E-FIC + VN01H1 Fab, Global refinement), EMD-73656 and PDB 9YYU (E-FIC + VN01H1 Fab, Fab

local refinement), EMD-73657 and PDB 9YYV (E-FIC + VN01H1 Fab, S2 local refinement), EMD-73787 and PDB 9Z3K (E-FICS-v1), EMD-75721 (E-FICS-v3 + VN01H1 Fab, Global refinement), EMD-75694 and PDB 000011HK (E-FICS-v3 + VN01H1 Fab, Fab local refinement), EMD-75695 and PDB 0000[illegible]11HL (E-FICS-v3 + VN01H1 Fab, S2 local refinement), EMD-75722 (E-FICS-v3 + C77G12 Fab, Global refinement), EMD-75705 and PDB 000011HW (E-FICS-v3 + C77G12 Fab, Fab local refinement), EMD-75697 and PDB 000011HN (E-FICS-v3 + C77G12 Fab, S2 local refinement). The mass spectrometry raw data and search results have been deposited to the ProteomeXchange Consortium via the PRIDE partner repository with the dataset identifier PXD069687.

Research involving human participants, their data, or biological material

Policy information about studies with human participants or human data. See also policy information about sex, gender (identity/presentation), and sexual orientation and race, ethnicity and racism.

Reporting on sex and gender	N/A
Reporting on race, ethnicity, or other socially relevant groupings	N/A
Population characteristics	N/A
Recruitment	N/A
Ethics oversight	N/A

Note that full information on the approval of the study protocol must also be provided in the manuscript.

Field-specific reporting

Please select the one below that is the best fit for your research. If you are not sure, read the appropriate sections before making your selection.

☒ Life sciences ☐ Behavioural & social sciences ☐ Ecological, evolutionary & environmental sciences

For a reference copy of the document with all sections, see nature.com/documents/nr-reporting-summary-flat.pdf

Life sciences study design

All studies must disclose on these points even when the disclosure is negative.

Sample size	Sample sizes were based on what is typically used in similar studies, along with our own prior experience. The number of replicates was sufficient to observe consistent and biologically meaningful differences. Key findings were confirmed across independent experiments to ensure reliability.
Data exclusions	No data were excluded from the analyses.
Replication	All key findings were independently replicated in at least two to three separate experiments using distinct technical and biological replicates. Results were consistent across replicates.
Randomization	Randomization was not necessary for this study due to the standardized nature of the experimental setup.
Blinding	Blinding was not performed in this study, as it was not relevant to the experimental design.

Reporting for specific materials, systems and methods

We require information from authors about some types of materials, experimental systems and methods used in many studies. Here, indicate whether each material, system or method listed is relevant to your study. If you are not sure if a list item applies to your research, read the appropriate section before selecting a response.

Materials & experimental systems

n/a	Involved in the study	
☐	☒	Antibodies
☐	☒	Eukaryotic cell lines
☒	☐	Palaeontology and archaeology
☐	☒	Animals and other organisms
☒	☐	Clinical data
☒	☐	Dual use research of concern
☒	☐	Plants

Methods

n/a	Involved in the study	
☒	☐	ChIP-seq
☒	☐	Flow cytometry
☒	☐	MRI-based neuroimaging

Antibodies

Antibodies used

Anti-SARS-CoV-2 S1 (MAB10540, R&D Systems), anti-SARS-CoV-2 S2 (humanized B6 IgG, described in PMID: 33981021, 10μg/mL and human 76E1, described in PMID: 35773398, 10μg/mL), anti-SARS-CoV-2 S2 polyclonal rabbit antibody (Sino Biological, 1:1000), donkey anti-mouse IgG 680 (LI-COR Biosciences), goat anti-human IgG 680 (LI-COR Biosciences), goat anti-rabbit IgG 680 (LI-COR Biosciences), anti-VSV nucleocapsid antibody (Absolute Antibody), goat anti-mouse IgG Alexa Fluor 647 or 488 (A-21235 or A-11001, Thermo Fisher Scientific), anti-SARS-CoV-2 nucleocapsid antibody (40143-R001, Sino Biological, 1:2000), and goat anti-rabbit IgG Alexa Fluor 647 (A-21245, 1:1000, Thermo Fisher Scientific).

Validation

Anti-SARS-CoV-2 S1 (MAB10540, R&D Systems, 2μg/mL) was validated by ELISA and Western blot against the RBD. Anti-SARS-CoV-2 S2 (humanized B6 IgG) was previously characterized (PMID: 33981021) and used here to detect S2 cleavage products. Anti-SARS-CoV-2 S2 polyclonal antibody (Sino Biological) was validated by the manufacturer for Western blot. Anti-VSV nucleocapsid (Absolute Antibody) is widely used in pseudovirus assays to detect VSV-N. Anti-SARS-CoV-2 nucleocapsid (40143-R001, Sino Biological) was validated for immunofluorescence by the manufacturer and used here to detect authentic virus infection.

Eukaryotic cell lines

Policy information about cell lines and Sex and Gender in Research

Cell line source(s)

Vero E6 cells (CRL-1586, ATCC) were maintained in Dulbecco's Modified Eagle Medium (DMEM) (Invitrogen) supplemented with 10% fetal bovine serum (FBS) (Omega Scientific) and 100 U/mL penicillin-streptomycin (P/S) (Invitrogen). Vero cells expressing TMPRSS2(70 or hACE2-TMPRSS2 (a gift of A. Creanga and B. Graham, National Institutes of Health (NIH)) were maintained as Vero E6 cells, with the addition of 5 μg/mL blasticidin (Vero E6-TMPRSS2) or 10 μg/mL of puromycin (Vero E6-hACE2-TMPRSS2). HEK293T (ATCC, CRL-3216) cells were cultured in DMEM (Gibco) supplemented with 10% FBS (Cytiva) and 1% penicillin-streptomycin (Life Tech). Calu-3 cells (ATCC, HTB-55) were maintained in DMEM/F-12 GlutaMAX™ supplement (Gibco) supplemented with 10% fetal bovine serum (FBS) (Cytiva). All cell lines were maintained at 37°C with 5% CO2. TASL-19 cells were previously described(87).

Authentication

Cell lines were not further authenticated.

Mycoplasma contamination

Cell lines were not tested for mycoplasma contamination.

Commonly misidentified lines
(See ICLAC register)

N/A

Animals and other research organisms

Policy information about studies involving animals; ARRIVE guidelines recommended for reporting animal research, and Sex and Gender in Research

Laboratory animals

This study involved laboratory mice, including hTMPRSS2 knock-in (C57BL/6J-Tmprss2em1(TMPRSS2)Synbl/J) and TMPRSS2 knockout (B6.129-Tmprss2tm1Psn/J) strains, as well as hTMPRSS2-KI mice (C57BL/6Hsd-Tmprss2em1(TMPRSS2)Env). Mice were between 5–8 weeks of age at the time of infection and were housed in HEPA filtered microisolator caging units up to 4 animals per cage at Bioqual. Environmental controls for the animal room were set to maintain 68°F to 79°F, a relative humidity of 30–70%, and a 12hr light/12hr dark cycle. Animals were housed in HEPA filtered microisolator caging units that contained up to 5 animals per cage at Washington University. Environmental conditions for the animal room were set to maintain temperature between 68°F to 74°F, a relative humidity of 30–60%, and a 12h light/12h dark cycle

Wild animals

N/A

Reporting on sex

Experimental groups included equal numbers of male and female mice to control for sex bias. Sex was considered during study design, and animals were age-matched across groups. However, sex-disaggregated data were not analyzed, as the study was not powered to detect sex-specific effects, and the primary outcomes were not expected to vary by sex.

Field-collected samples

N/A

Ethics oversight

Ethical approval was obtained for all experiments involving mice. These in vivo studies were conducted under protocols approved by

Ethics oversight	the Institutional Animal Care and Use Committee (IACUC) at Washington University and Bioqual. All other experiments involved recombinant protein expression and cell lines and did not require additional ethical oversight.

Note that full information on the approval of the study protocol must also be provided in the manuscript.

Plants

Seed stocks	N/A
Novel plant genotypes	N/A
Authentication	N/A
