# Supplementary figures and images for: TMPRSS2-mediated coronavirus spike activation and inhibition
Source: Nat Struct Mol Biol. 2026 Apr 28;33(5):810–23. doi: 10.1038/s41594-026-01801-y (PMC13186702; doi:10.1038/s41594-026-01801-y)

Raw Image

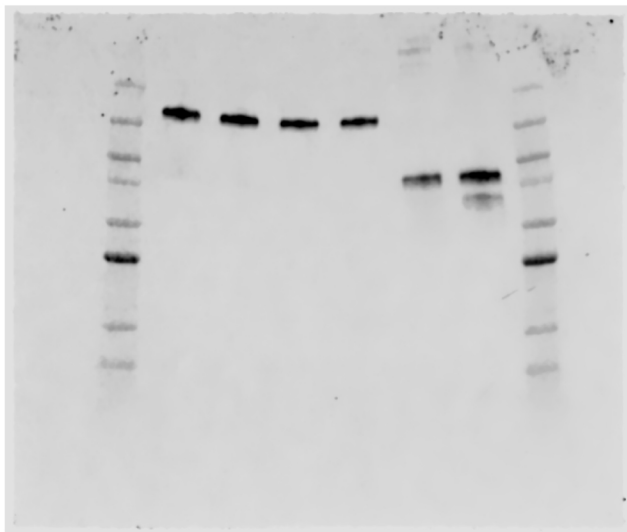

Figure 1c

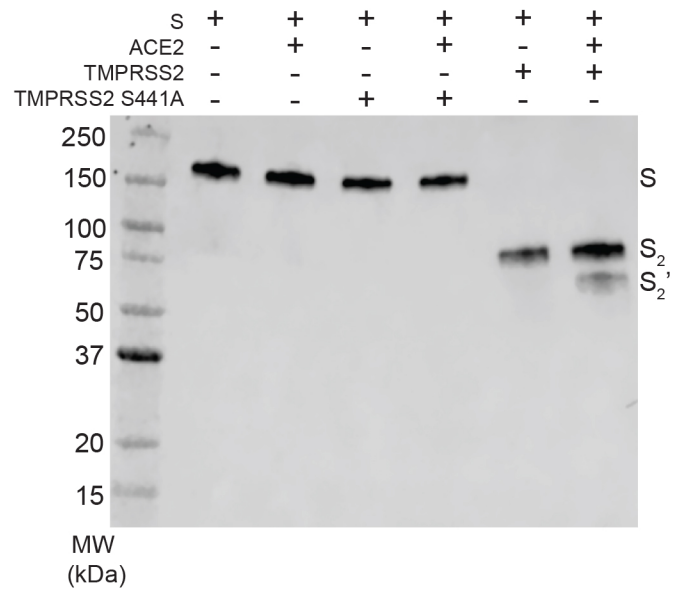

Raw Image

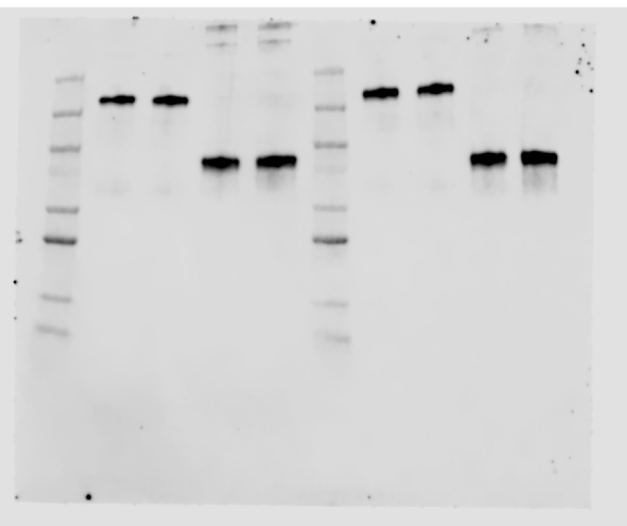

Figure 1g

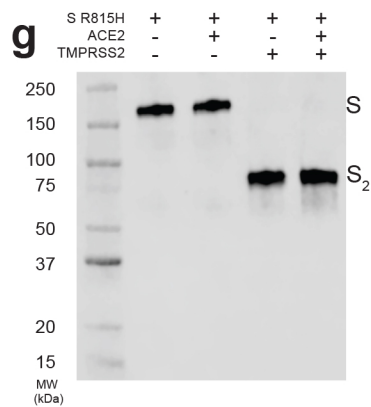

Supplement: Supplementary file 4 — Unprocessed western blots. [file 41594_2026_1801_MOESM4_ESM.pdf]

Raw image

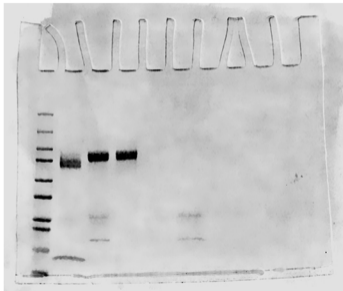

Fig3g

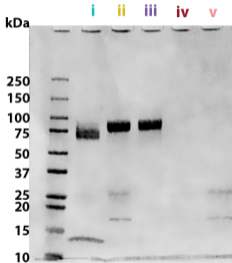

Supplement: Supplementary file 6 — Uncropped SDS–PAGE. [file 41594_2026_1801_MOESM6_ESM.pdf]
